# The t-SNARE protein FgPep12, associated with FgVam7, is essential for ascospore discharge and plant infection by trafficking Ca^2+^ ATPase FgNeo1 between Golgi and endosome/vacuole in *Fusarium graminearum*

**DOI:** 10.1371/journal.ppat.1007754

**Published:** 2019-05-08

**Authors:** Bing Li, Xin Dong, Rui Zhao, Rongchuan Kou, Xiaobo Zheng, Haifeng Zhang

**Affiliations:** 1 Department of Plant Pathology, College of Plant Protection, Nanjing Agricultural University, and Key Laboratory of Integrated Management of Crop Diseases and Pests, Ministry of Education, Nanjing, China; 2 College of Plant Protection, Nanjing Agricultural University, Nanjing, China; Purdue University, UNITED STATES

## Abstract

Soluble N-ethylmaleimide-sensitive factor attachment receptors (SNAREs) play a crucial role in the development and virulence through mediation of membrane fusion and vesicle trafficking in pathogens. Our previous studies reported that the SNARE protein FgVam7 and its binding proteins FgVps39/41 are involved in vesicle trafficking and are important for vegetative growth, asexual/sexual development, deoxynivalenol production and virulence in the Fusarium head blight fungus *Fusarium graminearum*. Here, we identified and characterized another FgVam7 binding protein in *F*. *graminearum*, FgPep12, an ortholog of yeast t-SNARE Pep12 with both the SNARE and TM domains being essential for its localization and function. Deletion of FgPep12 caused defects in vegetative growth, conidiogenesis, deoxynivalenol production and virulence. Cytological observation revealed that FgPep12 localizes to the Golgi apparatus, late endosomes and vacuoles, and is necessary for transport from the vacuole to prevacuolar compartment. Further investigation revealed that both FgPep12 and FgVam7 are essential for ascospore discharge through interaction with and trafficking of the Ca^2+^ ATPase FgNeo1 between the Golgi and endosomal/vacuolar system. FgNeo1 has similar biological roles to FgPep12 and is required for ascospore discharge in *F*. *graminearum*. Together, these results provide solid evidence to help unravel the mechanisms underlying the manipulation of ascospore discharge and plant infection by SNARE proteins in *F*. *graminearum*.

## Author summary

SNARE proteins which mediate fusion of transport vesicles with the correct target membrane, are essential components of vesicle trafficking machinery. Together with the cognate effectors, SNAREs coordinate the dynamics of trafficking pathway and determines the cargo proteins destination. Here, we found that SNARE protein FgPep12 is important for fungal development and virulence through its involvement in vesicle trafficking between the Golgi and endosomal/vacuolar system. We further provide multiple lines of evidence showing that SNARE proteins modulate development and ascospore discharge in pathogenic fungi. FgPep12, associated with FgVam7, is required for the trafficking of the Ca^2+^ ATPase FgNeo1 between the Golgi and endosomal/vacuolar system, thus controlling growth, asexual development, ascospore discharge and plant infection in *F*. *graminearum*.

## Introduction

In eukaryotic cells, directional transport among the organelles of the endomembrane system is mediated by vesicles that bud from a donor organelle and then fuse with an acceptor organelle [1]. Despite the diversity of these organelles in size and shape, the basic reactions of budding and fusion are mediated by multiprotein complexes consisting of protein families that have been conserved in eukaryotic organism [2–3]. One family of integral membrane proteins, called soluble N-ethylmaleimide-sensitive factor attachment receptor (SNARE) proteins, constitute the key machinery of these membrane fusion events [1] and have been studied extensively in budding yeast, mammals, plants, and phytopathogens [4–10]. Despite their differences in sizes and structures among organisms, SNAREs share a conserved SNARE motif of 60–70 amino acids (AAs) arranged in heptad repeats [4, 6, 11–12]. SNAREs can be classified into vesicle (v)-SNAREs and target (t)-SNAREs based on their localization to either vesicle membranes or target membranes [13]. Over 20 SNARE proteins have been identified in various species, including humans, *Drosophila melanogaster*, *Arabidopsis thaliana*, *Aspergillus oryzae*, and *Saccharomyces cerevisiae* [7–8, 14–16]. Similar to *A*. *oryzae*, 21 putative SNARE proteins have been identified in *Fusarium graminearum* [10]. In contrast, only a small number of SNARE proteins have been characterized in phytopathogens, including MoSec22, MoVam7, MoSyn8 and MoTlg2 in the rice blast fungus *Magnaporthe oryzae* [4, 6, 9, 17], UmYup1 in the corn smut fungus *Ustilago maydis* and FgVam7 in *F*. *graminearum* play critical roles in development, virulence and sexual reproduction by mediating vesicle trafficking [10, 18–19]. Additionally, t-SNARE MoSso1 is involved in virulence and is necessary for normal biotrophic interfacial complex development, as well as for secretion of cytoplasmic effectors during *M*. *oryzae* infection [20].

In some fungi of the phylum *Ascomycota*, ascospores formed within perithecia are forcibly discharged into the air, where they are believed to serve as the primary inoculum of the disease [21–24]. Sexual development and ascospore discharge are important factors in propagule dispersal, survival, and disease spreading in ascomycetes. Forcible discharge of ascospores from asci is a common mechanism observed in many fungi, including *F*. *graminearum* (teleomorph: *Gibberella zeae*), one of the casual agents of Fusarium head blight (FHB) or head scab, which is a destructive disease of wheat and barley [5, 25–26]. In *F*. *graminearum* and other fungal pathogens, ascospores are the primary inoculum, making sexual reproduction a critical step in the disease cycle. In addition to yield losses caused by FHB, mycotoxins such as deoxynivalenol (DON) and zearalenone (ZEA) produced by *F*. *graminearum* in contaminated grains pose a serious threat to human and animal health [27].

**Fig 1:**
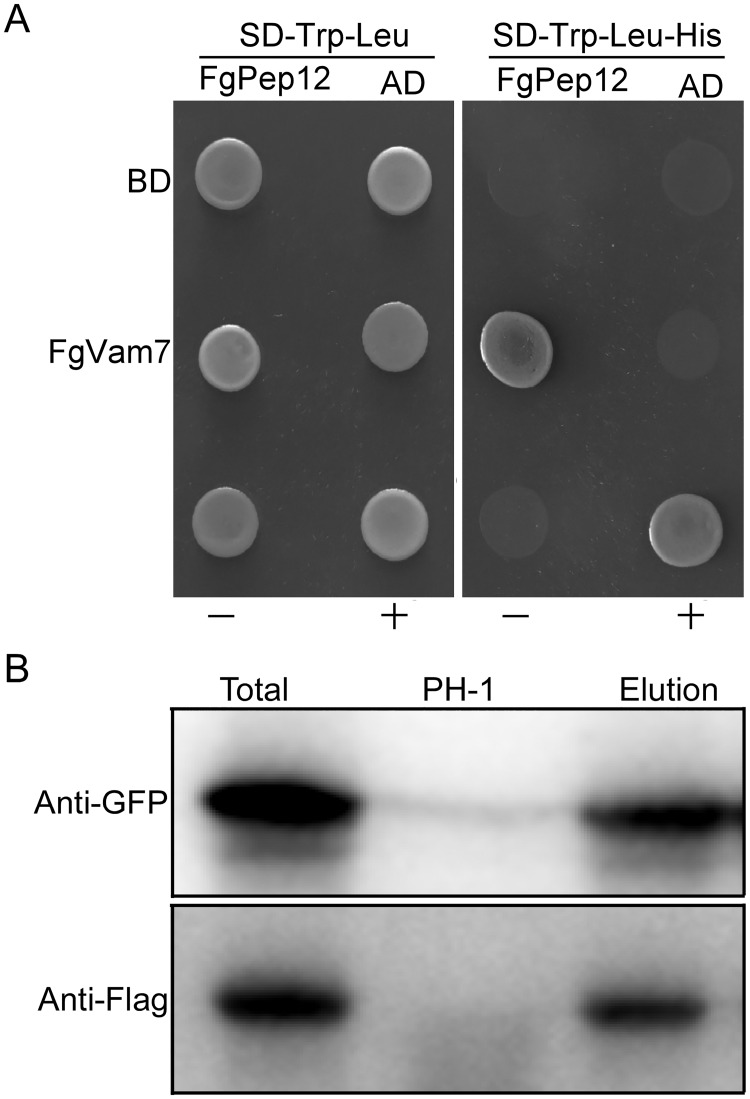
FgPep12 physically interacts with FgVam7. (A) Yeast two-hybrid assays for the interaction between FgPep12 and FgVam7. The prey and bait constructs were assayed for growth on SD-Leu-Trp and SD-Leu-Trp-His plates. +, positive control; -, negative control. (B) Co-immunoprecipitation assays for the interaction between FgPep12 and FgVam7. Total proteins were isolated from transformants co-expressing GFP-FgPep12 and FgVam7-3×FLAG constructs, and proteins eluted from the anti-FLAG M2 beads (elution). Immunoblots were incubated with monoclonal anti-FLAG and anti-GFP antibody, respectively.

Sexual reproduction in *F*. *graminearum* starts with the formation of small, coiled initials that eventually develop into perithecia filled with asci and ascospores that are the products of meiosis. Mature asci extend through the ostiole of the perithecia and discharge their ascospores [24, 28]. The main force driving discharge of ascospores is turgor pressure generated by ions and polyols in the asci [29]. In *F*. *graminearum*, potassium ion channel and L-type calcium channel blockers have been reported to inhibit ascospore discharge [22]. The accumulation of potassium and chloride ions generate the turgor pressure necessary for discharge [29]. Strains with defects in turgor pressure in the asci exhibited lower ion concentrations and failed to discharge ascospores [30]. Moreover, deletion of the calcium ion channel proteins Cch1 and Mid1 significantly reduced forcible ascospore discharge [30–31]. Meanwhile, deletion of Fig 1, a transmembrane protein in the low-affinity calcium uptake system, leads to failure of production of mature perithecia [32]. These reports demonstrate that ion channels, especially calcium ion channels, play crucial roles in ascospore discharge in *F*. *graminearum*.

Although previous studies have shed light on the physiological and genetic bases of ascospore discharge, the underlying mechanisms remain largely unknown, particularly how SNARE proteins modulate this process in *F*. *graminearum*. Here, we identified and characterized the t-SNARE FgPep12, and found that FgPep12 is important in development, DON production and virulence in *F*. *graminearum*. We further provide evidence that FgPep12 in association with another SNARE protein, FgVam7, is necessary for ascospore discharge based on trafficking of Ca^2+^ ATPase FgNeo1 between the Golgi apparatus and endosomal/vacuolar system.

## Results

### Identification and deletion of FgPep12 in *F*. *graminearum*

Our previous studies have shown that SNARE protein FgVam7 and its several binding proteins are involved in vesicle trafficking and play important roles in the development and virulence of *F*. *graminearum* [10]. Here, we identified another FgVam7 binding protein, FgPep12 (FGSG_01890: http://fungidb.org/fungidb/) by yeast two hybrid (Y2H) screening, which is an ortholog of the yeast t-SNARE Pep12 (S1 Fig). Y2H and co-immunoprecipitation (co-IP) assays confirmed that FgPep12 physically interacts with FgVam7 in *F*. *graminearum* (Fig 1A and 1B). The *FgPEP12* gene consists of 1,153 base pairs with two introns and encodes a 344-aa protein. Domain prediction revealed that FgPep12 possesses a SNARE domain (SNARE, 242–309 aas) and a transmembrane region (TM; 321–338 aas) at its carboxyl terminus (http://smart.emblheidelberg.de/). To explore the biological roles of FgPep12 in *F*. *graminearum*, we generated a gene replacement construct using a split marker strategy (S2A Fig). Southern blot analysis showed that *FgPEP12* was successfully replaced with the hygromycin B phosphotransferase (Hph) cassette in the Δ*Fgpep12* mutant (S2B Fig). The complemented transformant Δ*Fgpep12/FgPEP12* was generated by re-introducing the full-length *FgPEP12* sequence into the Δ*Fgpep12* mutant.

### FgPep12 is important for vegetative growth, conidiogenesis, pathogenesis and DON production

**Fig 2:**
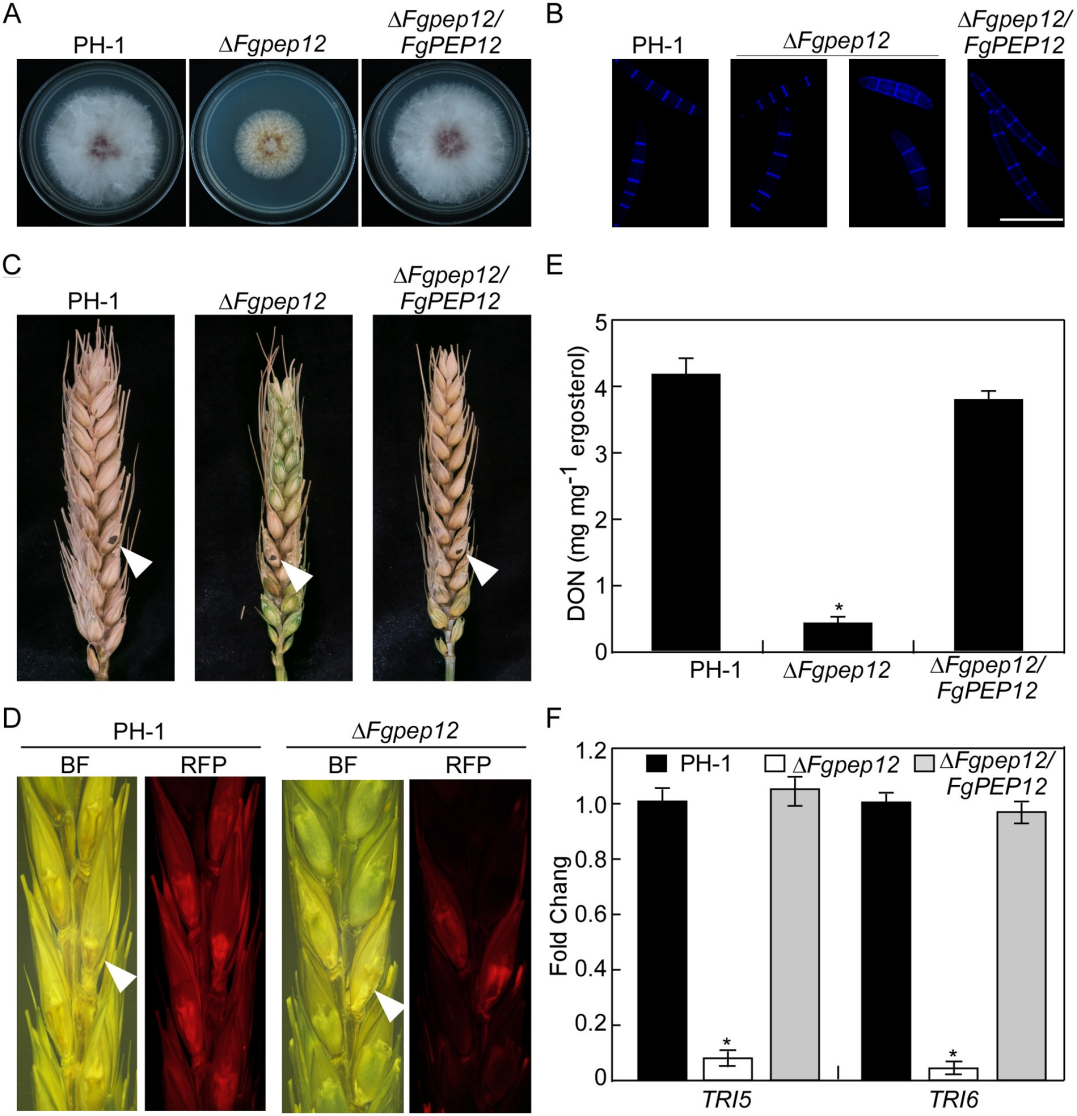
FgPep12 is important for vegetative growth, conidiogenesis, pathogenicity and DON production. (A) The wild type PH-1, Δ*Fgpep12* mutant and the complemented transformant Δ*Fgpep12/FgPEP12* were cultured on CM- media at 25°C for 3 days in the dark, and photographed. (B) Conidia of the indicated strains were harvested from CMC medium, and stained with calcofluor white, and observed under a fluorescence microscope. Bar = 10 μm. (C) Infection assays on flowering wheat heads. Conidia suspensions (10 μl) of PH-1, Δ*Fgpep12* and Δ*Fgpep12/FgPEP12* were injected into the flowering wheat heads. Photographs were taken at 14 days post-inoculation (dpi). Inoculated site were pointed by white arrows. (D) Spikelets were inoculated with conidia from PH-1 and Δ*Fgpep12* strain expressing *FgACTIN*-*RFP* construct. Images were taken at 7 dpi. Inoculated spikelets were indicated by white arrows. (E) DON production in wheat kernels infested by the indicated strains for 20 days. (F) Relative transcription abundance of trichothecene synthase genes *TRI5* and *TRI6* in the indicated strains. Error bars represent the SD and asterisks indicate statistically significant differences (*p*<0.01).

We first examined vegetative growth of the Δ*Fgpep12* mutant on CM and V8 juice agar plates. After 3 days of incubation in the dark at 25°C, the Δ*Fgpep12* mutant exhibited a smaller colony size and altered pigmentation compared to the wild-type PH-1 and the complemented strain Δ*Fgpep12/FgPEP12*. The colony diameter was reduced by 48.6% and 35.2% on CM and V8 plates, respectively (Fig 2A; Table 1). The Δ*Fgpep12* mutant produced very few conidia. Compared to the wild-type PH-1, conidiation was reduced over 90% (Table 1). Moreover, conidia produced by the Δ*Fgpep12* mutant were shorter in length and had fewer septa than those of PH-1 (Fig 2B; Table 1). These results indicate that FgPep12 plays important roles in vegetative growth and conidiogenesis in *F*. *graminearum*.

**Table 1:** Phenotype analysis of the PH-1, Δ*Fgpep12*, Δ*Fgpep12/FgPEP12* and domain deletion strains.

Strain	Colony diameter (cm)^a^		Conidiation	Conidial morphology (%)^c^		Conidial length (μm)^d^	Number of perithecia (cm^2^)^e^
	CM	V8	(×10^6^/ml)^b^	≥4 Septa	≤3 Septa		
PH-1	7.4 ± 0.4	5.4 ± 0.1	12.6 ± 0.8	68.3 ± 2.1	31.7 ± 2.2	46.16 ± 3.20	69.6 ± 3.2
Δ*Fgpep12*	3.8 ± 0.1*	3.5 ± 0.2*	0.6 ± 0.09*	43.2 ± 1.7*	56.8 ± 2.6*	27.18 ± 2.86*	66.9 ± 5.9
ΔSNARE	3.8 ± 0.1*	NA	0.6 ± 0.08*	40.1 ± 2.3*	59.9 ± 1.4*	28.53 ± 3.48*	NA
ΔTM	3.6 ± 0.3*	NA	0.8 ± 0.03*	45.2 ± 3.8*	54.8 ± 3.1*	25.68 ± 1.86*	NA
Δ*Fgpep12/FgPEP12*	7.2 ± 0.2	5.5 ± 0.33	13.1 ± 0.4	66.9 ± 4.6	33.1 ± 2.5	47.23 ± 2.80	70.1 ± 1.2

^a^ Colony diameter of the indicated strains on different media after 3 days incubation at 25°C.

^b^ Quantification of the conidial production of the indicated strains from CMC cultures.

^c^ Percentage of the abnormal conidia of the indicated strains.

^d^ Measurement of the conidial length of the indicated strains.

^e^ The number of perithecia of the indicated strains on carrot agar and then induced by 2.5% Tween-60 at 18°C.

±SD was calculated from three repeated experiments and asterisks indicate statistically significant differences (*p*<0.01).

NA: not assayed.

We subsequently investigated the involvement of FgPep12 in virulence, by inoculating conidial suspensions of PH-1, Δ*Fgpep12* and Δ*Fgpep12/FgPEP12* on flowering wheat heads, as described previously [10]. Two weeks after inoculation, the Δ*Fgpep12* mutant only caused necrotic symptoms in the inoculated and nearby spikelets, compared to infection of the entire wheat heads by the wild-type PH-1 and complemented strain (Fig 2C). To confirm this observation, flowering wheat heads were inoculated with the wild-type PH-1 and Δ*Fgpep12* mutant expressing the *FgACTIN*-RFP construct. The red fluorescent protein (RFP) signal was observed near the inoculation site of the mutant, whereas it spread from the inoculation site to the entire wheat head in samples inoculated with PH-1 (Fig 2D). DON is known to act as a virulence factor in *F*. *graminearum* [33–35]. Therefore, we measured DON production of PH-1 and the Δ*Fgpep12* mutant as described previously [36], and found that DON production was significantly reduced in the mutant, with only 10% of the wild-type level (Fig 2E). The expression levels of two trichothecene biosynthesis genes, *TRI5* and *TRI6* [37], were notably reduced in the Δ*Fgpep12* mutant compared to PH-1 and complemented strain (Fig 2F). These findings suggest that FgPep12 is important for virulence and DON production in *F*. *graminearum*.

### FgPep12 localizes to Golgi apparatus, late endosomes and vacuoles

**Fig 3:**
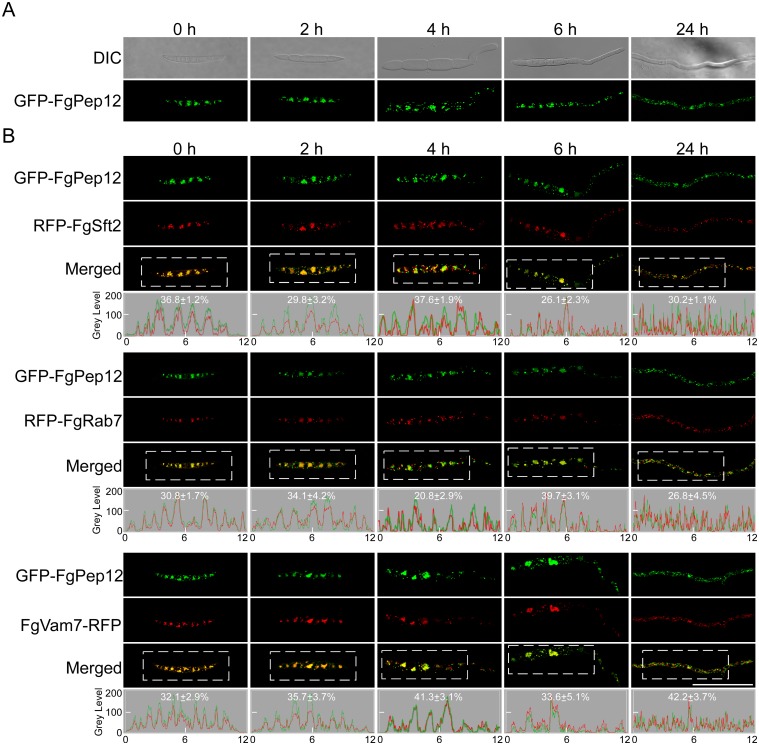
FgPep12 localizes to Golgi, late endosome and vacuole multiple organelles. (A) Expression and localization of GFP-FgPep12 in *F*. *graminearum*. Conidia expressing GFP-FgPep12 were germinated in liquid CM medium for 0, 2, 4, 6 and 24 h, and examined under differential interference contrast (DIC) and epifluorescence microscope. (B) Conidia co-expressing GFP-FgPep12&RFP-FgSft2, GFP-FgPep12&RFP-FgRab7 or GFP-FgPep12&FgVam7-RFP constructs were germinated in liquid CM medium for 0, 2, 4, 6 and 24 h, and examined under a confocal microscope. Co-localization of the proteins was further evaluated by linescan graph analysis (grey panel, horizontal axis indicates the distance). The analyzed area was indicated in dashed box. The percentage of the localization signals was yielded by counted the merged dots in conidia (n = 30) or germ tubes (n = 30, per germ tube 10 μm). Bar = 10 μm.

To determine the function of FgPep12, we fused a green fluorescent protein (GFP) tag to the N-terminus of FgPep12 and examined its cellular localization. The GFP-FgPep12 signals were visible in punctate structures throughout the cell (Fig 3A). In yeast, Pep12 was known to act as a vesicular intermediate, traveling between Golgi apparatus and the vacuole [3]. Therefore, we co-expressed GFP-FgPep12 with the Golgi marker protein FgSft2, which is a homolog of yeast Sft2 [38], the late endosome marker protein FgRab7 [39], and the vacuole localization protein FgVam7 fused to RFP [10] in *F*. *graminearum*. In the resulting transformants, the GFP-FgPep12 signal overlapped well with RFP-FgSft2, RFP-FgRab7 and FgVam7-RFP in conidia and germ tubes (Fig 3B). These co-localization patterns were further evaluated through linescan graph analysis. The percentage of GFP-FgPep12 co-localization with RFP-FgSft2, RFP-FgRab7 and FgVam7-RFP was between 20.8 and 42.2% in conidia and germ tubes (Fig 3B). These results indicated that, similar to yeast Pep12, FgPep12 likely functions as a vesicular intermediate between the Golgi apparatus and vacuole in *F*. *graminearum*.

### The SNARE and TM domains of FgPep12 are required for its cellular localization and biological function

**Fig 4:**
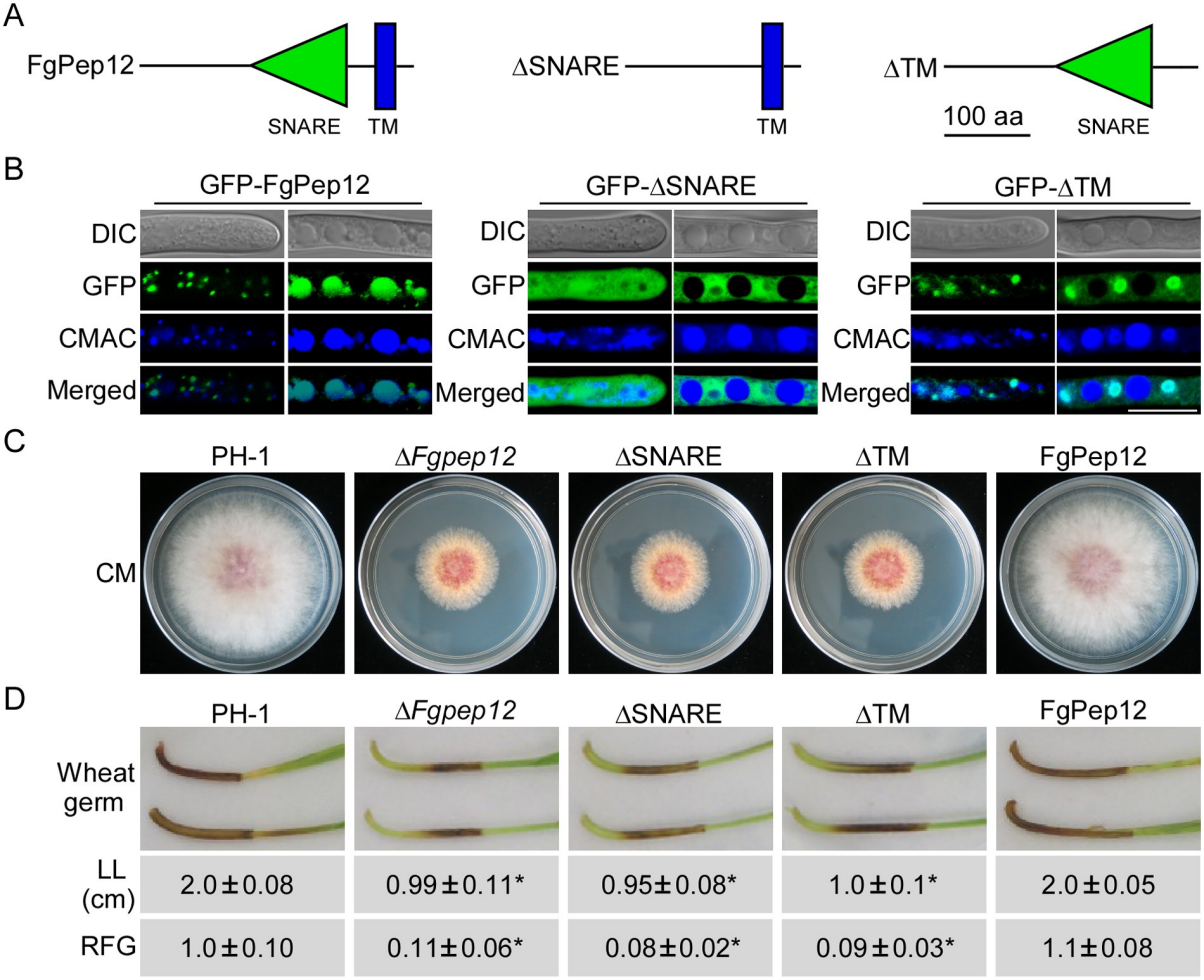
The TM and SNARE domains of FgPep12 are required for its cellular localization and biological function. (A) Diagram of FgPep12 and domain deletion strategy. (B) Subcellular localization of domain deletion proteins. Bar = 10 μm. (C) Vegetative growth and colony morphology of the domain deletion transformants. (D) Infection assay of the domain deletion transformants. Wheat germs were drop-inoculated with conidial suspensions of the indicated strains. Lesion length (LL) was measured at 10 dpi and photographed. Relative fungal growth (RFG) was analyzed by quantification of *F*. *graminearum* 28s rDNA relative to wheat genomic *TaACTIN* DNA. ±SD was calculated from three independent experiments, and asterisks indicate statistically significant differences (*p*<0.01).

To explore the role of the SNARE and TM domains of FgPep12, the domain deletion constructs ΔSNARE and ΔTM were generated (Fig 4A) and transformed into the Δ*Fgpep12* mutant. The resulting transformants GFP-ΔSNARE and GFP-ΔTM were obtained and phenotypically analyzed. Compared to GFP-FgPep12, the localization patterns of both GFP-ΔSNARE and GFP-ΔTM were altered. GFP-ΔSNARE mainly localized to the cytoplasm outside of vacuoles, and GFP-ΔTM mainly to pre-vacuole structures near mature vacuoles (Fig 4B). When assayed for growth, conidiation and virulence, both GFP-ΔSNARE and GFP-ΔTM transformants had similar phenotypes to those of the Δ*Fgpep12* mutant (Fig 4C and 4D; Table 1). These findings suggested that the SNARE and TM domains are required for proper localization and normal function of FgPep12 in *F*. *graminearum*.

### FgPep12 is required for vacuole to prevacuolar compartment (PVC) transport

**Fig 5:**
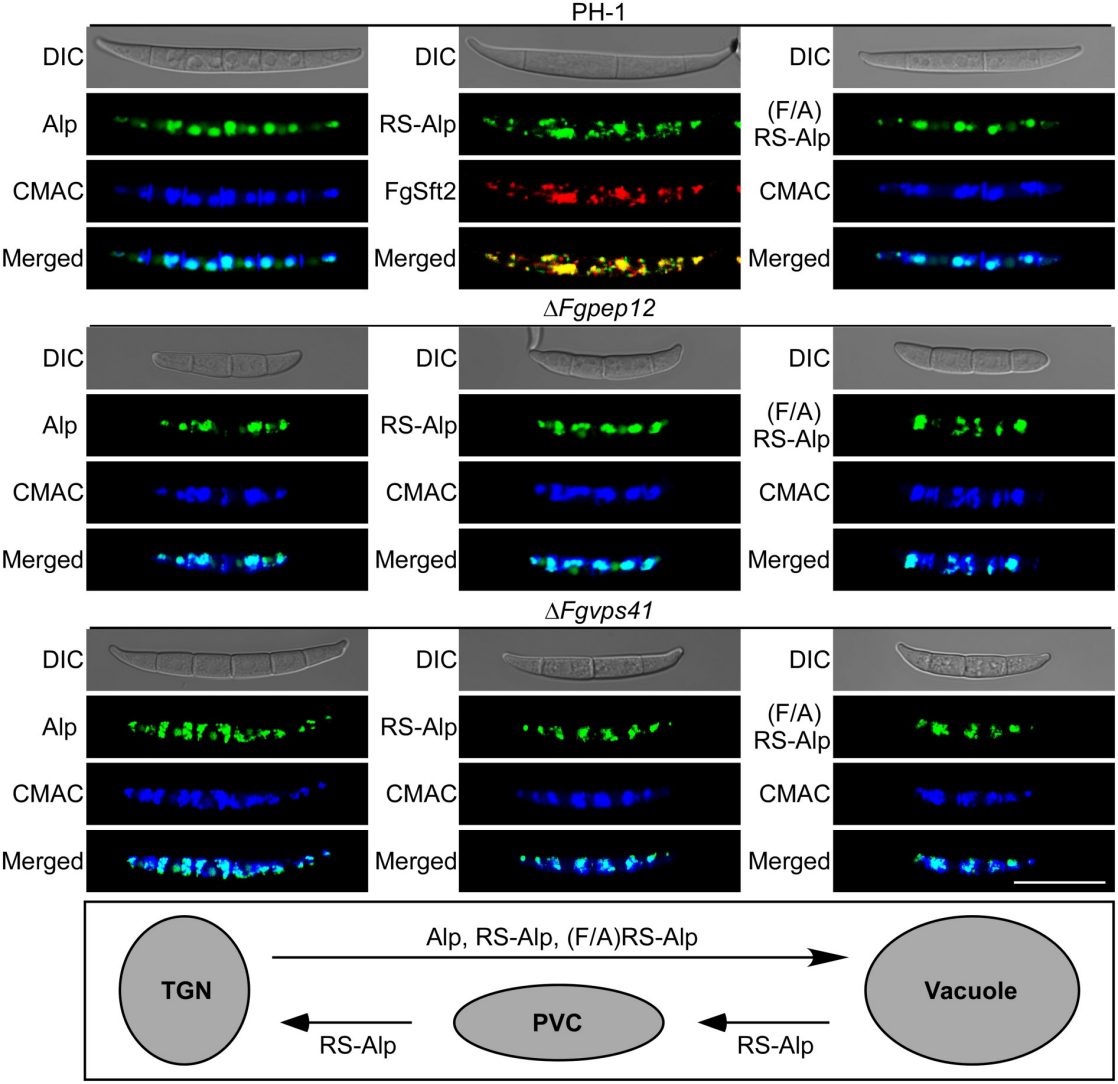
FgPep12 is required for retrograde transport of RS-Alp from the vacuole to the PVC. Conidia of transformants expressing Alp-GFP, RS-Alp-GFP and (F/A)RS-Alp-GFP in the wild type PH-1, Δ*Fgpep12* and Δ*Fgvps41* mutants were examined by DIC or fluorescence microscope. CMAC staining or expressing RFP-FgSft2 was performed to co-localize with target proteins. Bar = 10 μm.

Pep12 has been reported to play a role in retrograde transport out of the vacuole based on the unique properties of RS-ALP and (F/A)RS-ALP in yeast [40–41]. RS-ALP contains the Golgi retrieval motif (FXFXD) fused to the cytosolic domain of ALP, which can be retrograde-transported to the Golgi via the PVC [40]. Meanwhile, both phenylalanine residues in the FXFXD retrieval motif of (F/A)RS-ALP were mutated to alanine, and thus would be trapped on the vacuolar membrane [42]. To investigate whether FgPep12 plays a similar role in *F*. *graminearum*, we generated yeast Alp-GFP, RS-Alp-GFP and (F/A)RS-Alp-GFP constructs and transformed them into the PH-1 and Δ*Fgpep12* mutants. The resulting transformants with green fluorescence were screened and selected for localization observation. Both Alp-GFP and (F/A)RS-Alp-GFP localized to vacuoles in the wild-type PH-1, while RS-Alp-GFP localized to Golgi organelles as in yeast (Fig 5). Similar to the results for the wild type, Alp-GFP and (F/A)RS-Alp-GFP localized to vacuoles in the Δ*Fgpep12* mutant, but RS-Alp-GFP was accumulated and localized in vacuoles in the mutant (Fig 5), indicating that FgPep12 plays a role in retrograde transport of RS-Alp-GFP from the vacuole to Golgi. To determine whether this transport occurs via the PVC pathway, we further obtained transformants expressing Alp-GFP, RS-Alp-GFP and (F/A)RS-Alp-GFP constructs in the Δ*Fgvps41* mutant. FgVps41 is a vacuolar sorting protein that mediates membrane fusion and PVC-to-vacuole trafficking in *F*. *graminearum* [43]. Microscopic observation revealed that GFP signals of Alp-GFP, RS-Alp-GFP and (F/A)RS-Alp-GFP accumulated and localized to vacuoles in the Δ*Fgvps41* mutant (Fig 5). The results suggest that FgPep12 is indispensable for retrograde transport of RS-Alp from the vacuole to the PVC.

### FgPep12 and FgVam7 are required for ascospore discharge in *F*. *graminearum*

**Fig 6:**
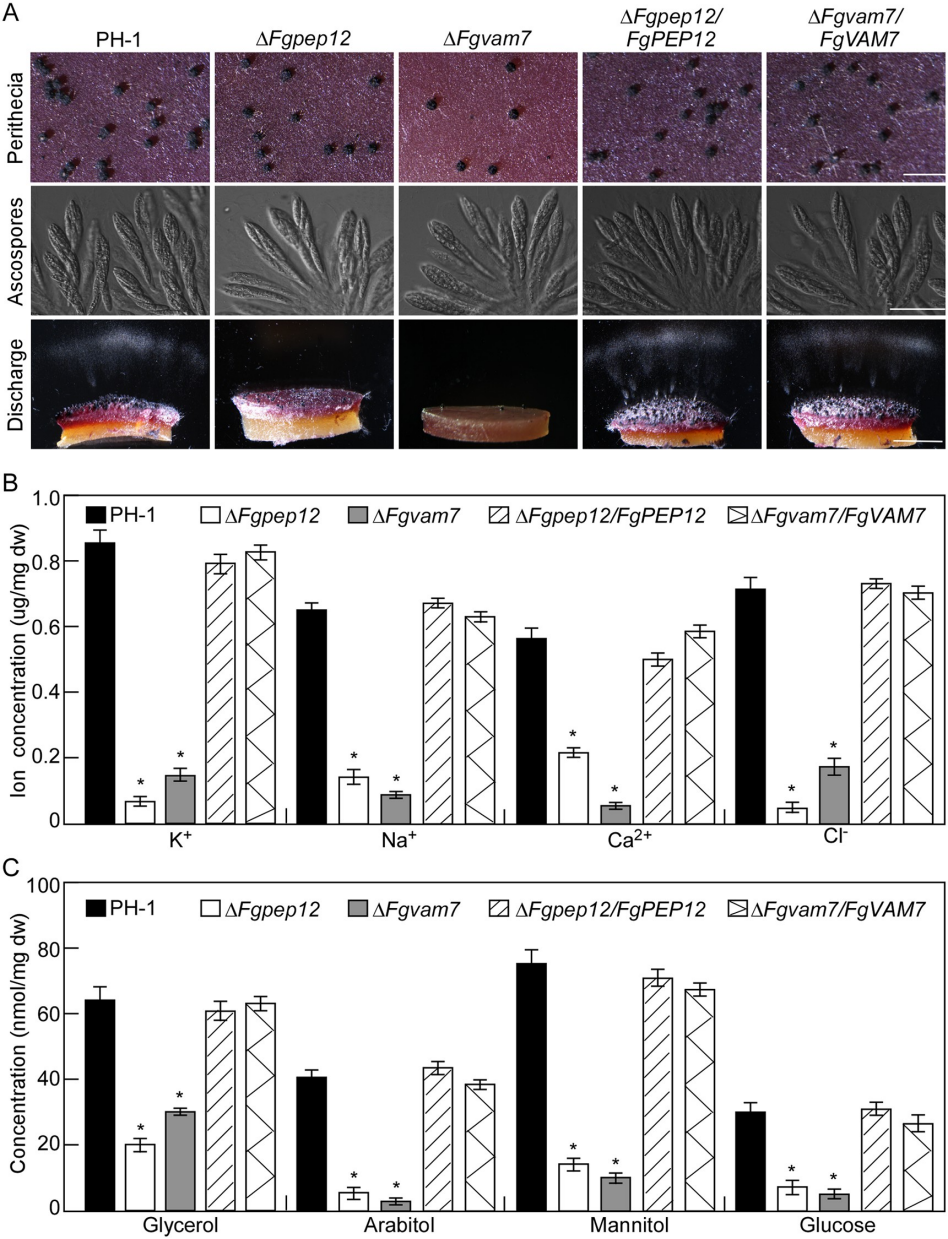
FgPep12 and FgVam7 are essential for ascospore discharge, and for maintaining the normal concentration of ions and polyols in asci. (A) Perithecia, asci formation and ascospore discharge of the indicated strains on carrot agar plates, and photographed at 10 dpi. Bar = 1 mm. The concentration of ions (B) and polyols (C) in asci of the indicated strains was measured by spectrophotometrically, respectively. Error bars represent the SD and asterisks indicate statistically significant differences (*p*<0.01).

The perithecia and ascospores of *F*. *graminearum* play a critical role in its disease cycle [44]. Therefore, we assessed sexual reproduction of the Δ*Fgpep12* and Δ*Fgvam7* mutants on carrot agar plates. Ten days after inoculation, the Δ*Fgpep12* mutant produced a large number of mature perithecia and asci, similar to the wild-type PH-1 (Fig 6A), while the Δ*Fgvam7* mutant produced fewer perithecia, consistent with the results of our previous study [10]. When assayed for ascospore release, no ascospores were discharged from the perithecia of the Δ*Fgvam7* and Δ*Fgpep12* mutants, in contrast to the numerous ascospores discharged from the wild-type PH-1 (Fig 6A). These results suggested that FgVam7 and FgPep12 play indispensable roles in ascospore discharge in *F*. *graminearum*. Because the turgor pressure generated by different ions and polyols in the asci is responsible for ascospore discharge in *F*. *graminearum* [22, 24, 30–32], we speculate that the defect of ascospore discharge in the Δ*Fgvam7* and Δ*Fgpep12* mutants is likely related to defects in turgor pressure generation. Therefore, we measured the concentrations of various ions and polyols in the asci of the Δ*Fgvam7* and Δ*Fgpep12* mutants. Compared to the wild-type PH-1 and complemented strains, the concentrations of ions (K^+^, Cl^-^, Ca^2+^ and Na^+^) and polyols (glycerol, arabitol, mannitol and glucose) were significantly lower in both mutants (Fig 6B and 6C), suggesting that the Δ*Fgvam7* and Δ*Fgpep12* mutants possessed low turgor pressure in the asci and therefore failed to discharge ascospores.

### Ion channel impairment results in arrest of ascospore discharge in *F*. *graminearum*

Ion channels are involved in ion buildup, which drives the influx of water and causes turgor pressure, which stretches the asci [29]. To reveal the underlying role of ion channels in ascospore discharge in *F*. *graminearum*, perithecia of the wild-type PH-1 were treated with ion channel inhibitors and their ascospore discharge was evaluated. We found that blocking K^+^, Ca^2+^ and Cl^-^ channels with inhibitors (K^+^: CsCl or glyburide; Ca^2+^: TMB8; Cl^-^: DIDS or 9-AC) significantly reduced ascospore discharge by *F*. *graminearum*. The reduction rate was 40–60% when K^+^ or Cl^-^ channels were blocked, and over 99% when Ca^2+^ channels were blocked (Table 2), suggesting that ion channels indeed play an important role in ascospore discharge by *F*. *graminearum*. We further analyzed ascospore discharge in the Δ*Fgvam7* and Δ*Fgpep12* mutants through treatment with exogenous ions. The ascospore discharge defect of the Δ*Fgvam7* and Δ*Fgpep12* mutants was greatly rescued by treatment with K^+^, Na^+^, Cl^-^ and Ca^2+^. The number of discharged ascospores increased to over 31% in Δ*Fgvam7* and over 45% in Δ*Fgpep12*, as compared to the wild-type PH-1 (Table 3). Together, these results suggest that ion channel impairment results in arrest of ascospore discharge in *F*. *graminearum*.

**Table 2:** Inhibition rate of ascospore discharge of PH-1 under various ionic inhibitors treatment.

Inhibitor	Compound^a^	Concentration	Inhibition Rate (%)
K^+^ inhibitor	CsCl	5.0 mM	53.6 ± 2.3
	Glyburide	0.5 mM	47.3 ± 3.8
Ca^2+^ inhibitor	TMB8	0.5 mM	80.9 ± 2.8
		1.0 mM	99.3 ± 3.0
Cl^-^ inhibitor	DIDS	50 μM	61.3 ± 1.6
	9-AC	20 μM	52.1 ± 4.2
CK	Ethanol	0.5 mM	0.3 ± 0.1
CK	HCl	0.5 mM	1.2 ± 0.6

±SD was calculated from three independent experiments. CK: control (dissolvant).

**Table 3:** Ascospore discharge of the indicated strains under various ions treatment.

Strains	Compounds														
	Water Agar	NH_4_NO_3_		KNO_3_		NaNO_3_		KCl		NaCl		NH_4_Cl		CaCl_2_	
		0.5 mM	1.0 mM	0.5 mM	1.0 mM	0.5 mM	1.0 mM	0.5 mM	1.0 mM	0.5 mM	1.0 mM	0.5 mM	1.0 mM	0.5 mM	1.0 mM
PH-1	139.6 ± 3.4	134.3± 0.6	140.6 ± 6.2	143.6 ± 3.6	137.3 ± 6.1	139.7 ± 2.1	136.7 ± 3.9	128.6 ± 5.4	135.6 ± 4.8	129.5 ± 6.5	137.5 ± 3.7	124.8 ± 6.7	136.6 ± 4.4	127.7 ± 1.3	141.7 ± 6.3
Δ*Fgpep12*	0	0	0	67.3 ± 3.6*	82.1 ± 0.6*	63.6 ± 2.5*	79.6 ± 2.5*	74.6 ± 6.4*	92.1 ± 2.1*	69.6 ± 1.9*	87.8 ± 2.1*	56.1 ± 2.6*	76.1 ± 4.2*	70.8 ± 4.6*	93.8 ± 7.6*
Δ*Fgvam7*	0	0	0	45.2 ± 2.8*	45.2 ± 2.8*	53.4 ± 1.7*	63.4 ± 1.7*	61.8 ± 3.1*	84.2 ± 1.5*	71.4 ± 3.7*	91.1 ± 0.2*	63.4 ± 6.2*	69.9 ± 6.1*	65.4 ± 2.1*	86.8 ± 3.4*
Δ*Fgpep12*/ *FgPEP12*	131.8 ± 5.2	127.1± 4.6	138.2 ± 3.9	139.1 ± 7.2	143.7 ± 5.8	142.8 ± 4.2	138.7 ± 4.8	138.6 ± 7.4	127.6 ± 6.6	134.5 ± 4.9	141.9 ± 6.7	132.1 ± 4.1	124.1 ± 0.7	131.9 ± 0.7	139.2 ± 2.3
Δ*Fgvam7*/ *FgVAM7*	143.1 ± 2.1	134.1± 4.6	136.8 ± 1.7	141.1 ± 3.8	133.1 ± 3.3	135.8 ± 6.2	129.8 ± 6.2	135.6 ± 4.1	137.6 ± 1.3	138.5 ± 5.5	130.5 ± 1.9	129.4 ± 7.1	135.2 ± 2.8	134.1 ± 3.1	146.7 ± 8.1

Each number indicates the ascospores discharged from 35 perithecia. ±SD was calculated from three independent experiments, and asterisks indicate statistically significant differences (*p*<0.01).

### Both FgVam7 and FgPep12 physically interact with Ca^2+^ ATPase FgNeo1 in *F*. *graminearum*

**Fig 7:**
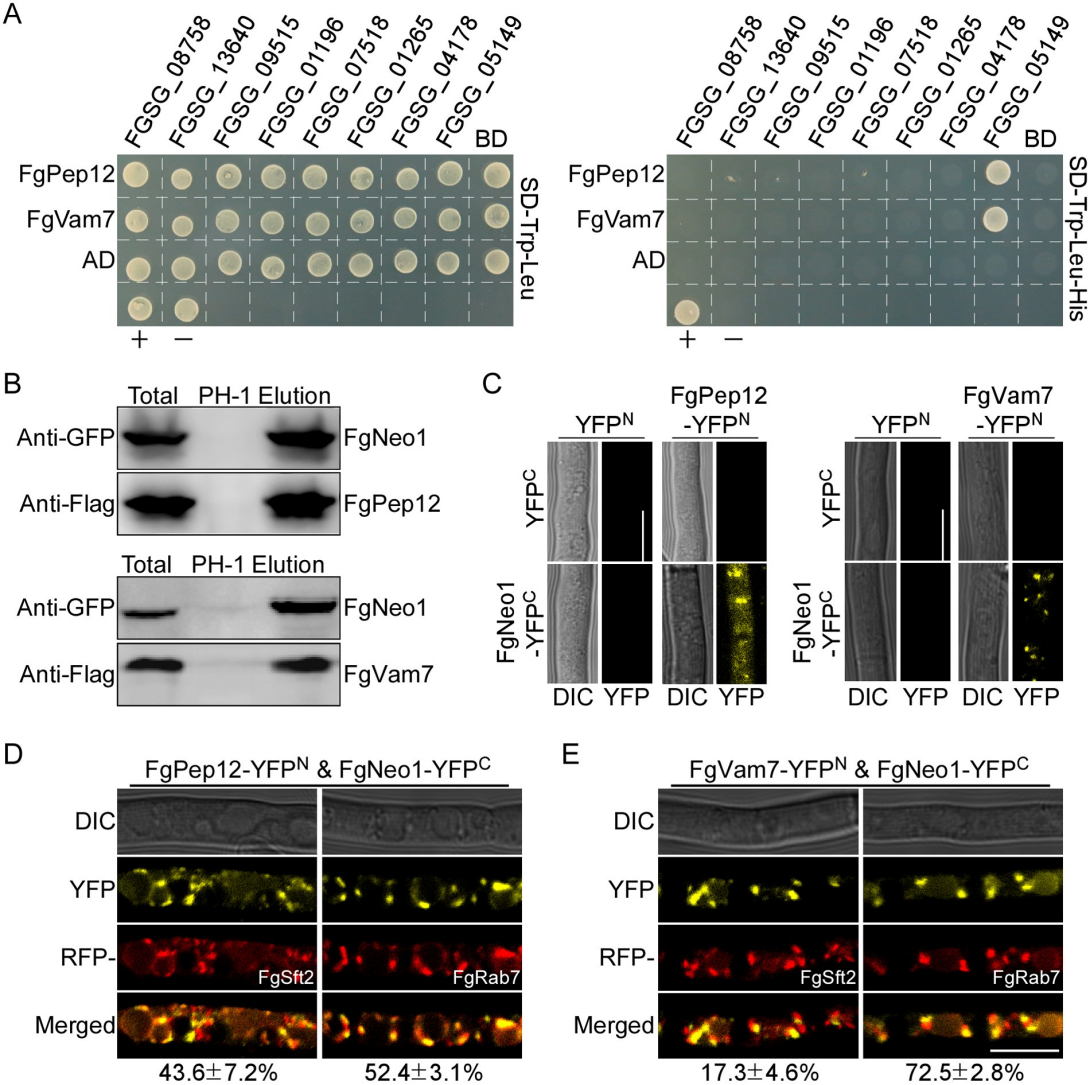
FgPep12 and FgVam7 physically interact with Ca^2+^ ATPase FgNeo1 in *F*. *graminearum*. (A) Y2H assays for the interactions between FgPep12, FgVam7 and the predicted intracellular calcium signaling-related proteins, respectively. +, positive control; -, negative control. (B) Co-IP assays for the interactions between FgPep12 and FgNeo1, FgVam7 and FgNeo1. Total proteins were isolated from transformants co-expressing GFP-FgNeo1 and FgVam7-3×FLAG or FgPep12-3×FLAG constructs, and proteins eluted from the anti-FLAG M2 beads (elution). Immunoblots were incubated with monoclonal anti-FLAG and anti-GFP antibody, respectively. (C) BiFC assays for the interactions between FgPep12 and FgNeo1, FgVam7 and FgNeo1, respectively. (D and E) Hyphae of transformants expressing RFP-FgSft2 or RFP-FgRab7 constructs in the FgPep12&FgNeo1 and FgVam7&FgNeo1 BiFC strains, were examined under a confocal microscope. Numbers indicate the merged rate of YFP and RFP proteins. Bar = 10μm.

Both FgVam7 and FgPep12 localized to intracellular compartments, and the Ca^2+^ channel is likely the most important ion channel for ascospore discharge based on the results described above. To determine the relationship between Ca^2+^ channel proteins and FgVam7 or FgPep12, eight intracellular calcium signaling-related proteins with predicted functions as Ca^2+^ pumps, Ca^2+^ exchangers and Ca^2+^ permeable channels were identified in *F*. *graminearum* based on the model of calcium signaling and transport pathways in the rice blast fungus [45]. We first analyzed the interactions between FgVam7, FgPep12 and these eight proteins using Y2H assays. Both FgVam7 and FgPep12 physically interact with the putative Ca^2+^ pump protein Ca^2+^ ATPase FgNeo1 (FGSG_05149), but not with the other seven proteins assessed in the Y2H assays (Fig 7A). The interactions among FgVam7, FgPep12 and FgNeo1 were confirmed by co-IP and bimolecular fluorescence complementation (BiFC) assays (Fig 7B and 7C). Combined with the interaction observed between FgVam7 and FgPep12, we propose that FgNeo1, FgPep12 and FgVam7 interact with each other to form a complex in *F*. *graminearum*. In the FgPep12&FgNeo1 and FgVam7&FgNeo1 BiFC transformants, YFP signals were observed in punctate structures (Fig 7C). To identify these structures, we expressed RFP-FgSft2 and RFP-FgRab7 in the two BiFC transformants and obtained RFP transformants. Fluorescence observation revealed that interactions between FgPep12 and FgNeo1 mainly occurred in the Golgi apparatus (43.6%) and late endosomes (52.4%) (Fig 7D). Interactions between FgVam7 and FgNeo1 mainly occurred in late endosomes (72.5%) but also sometimes occurred in the Golgi apparatus (17.3%) (Fig 7E). Similar results were also observed in ascospores in these two BiFC transformants (S3A and S3B Fig). In addition, we found FgPep12&FgNeo1 and FgVam7&FgNeo1 displayed dynamic mobility and also merged with Golgi apparatus and late endosomes in hyphae (S1–S4 Videos). These results indicate that FgPep12 and FgVam7 likely mediate FgNeo1 trafficking between the Golgi and endosomal/vacuolar system in *F*. *graminearum*.

### FgNeo1 is involved in vegetative, asexual, and sexual development and virulence in *F*. *graminearum*

**Fig 8:**
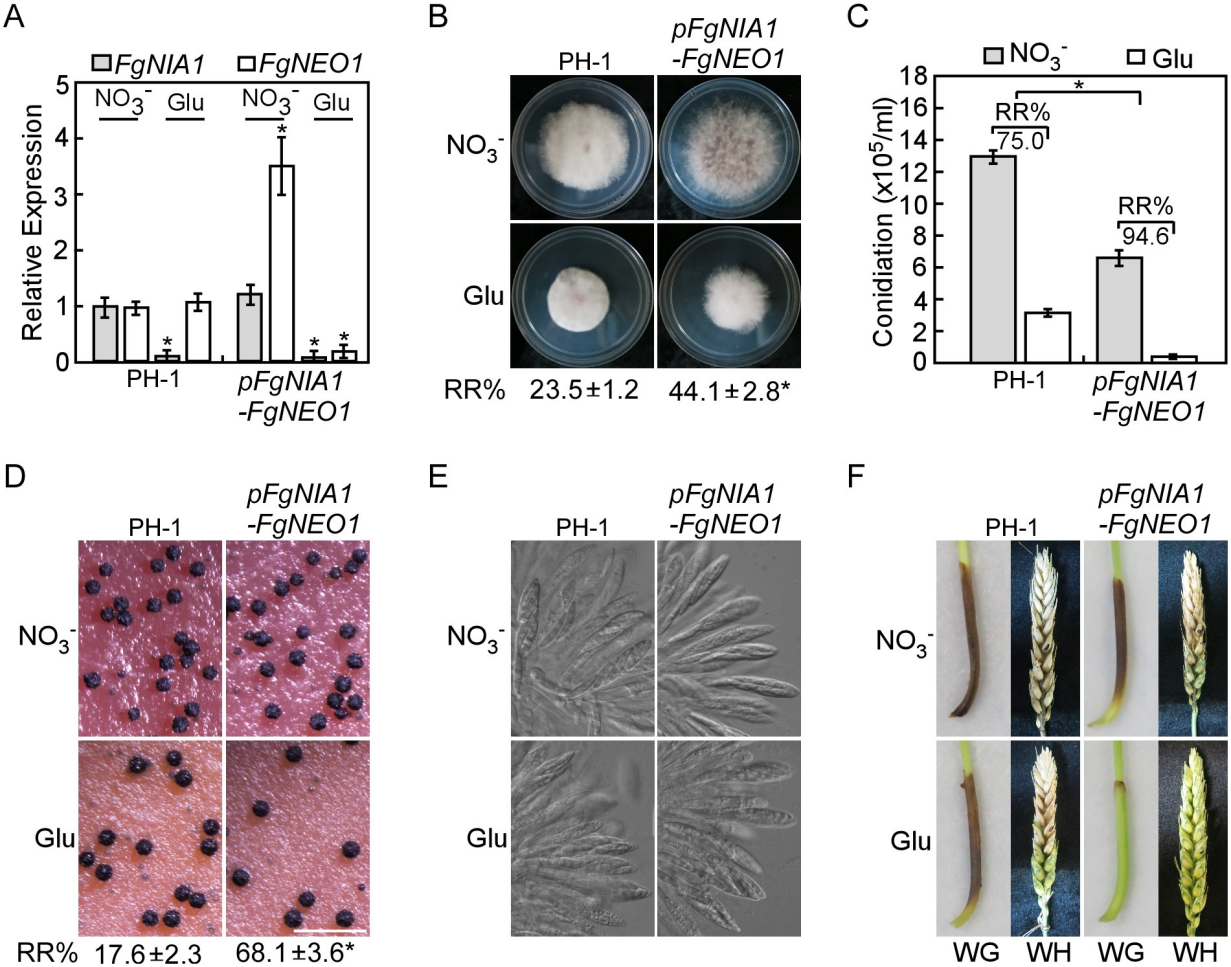
FgNeo1 is involved in vegetative growth, asexual/sexual development and virulence. (A) Quantitative RT-PCR monitoring of *FgNIA1* and *FgNEO1* expression in the wild type PH-1 and transformant *pFgNIA1-FgNEO1* under the control of *pFgNIA1*. The strains were cultured in MM+NO_3_^-^ and MM+Glu, respectively. (B) Colony morphology of the indicated strains on different media, and statistical analysis of the percentage of relative reduction (RR) in growth. (C) Quantification and analysis of the percentage of relative reduction in conidial production. (D and E) Perithecia, asci formation and morphology of the indicated strains. Bar = 1 mm. (F) Conidial suspensions of the indicated strains with NO_3_^-^ or Glu, were drop-inoculated onto wheat germs (WG) or injected into the flowering wheat heads (WH). Photographs were taken at 10 dpi and 14 dpi, respectively. Error bars represent the SD and asterisks indicate statistically significant differences (*p*<0.01).

Because FgNeo1, FgPep12 and FgVam7 interact with each other, these three proteins may regulate similar biological processes. Sequence analysis revealed that FgNeo1 is a putative homolog of yeast Neo1, a phospholipid-translocating ATPase (Ca^2+^ATPase) that is essential in yeast [46]. Therefore, we adopted the conditional promoter replacement (CPR) approach for inducing (NaNO_3_: NO_3_^-^) or silencing (Glutamate: Glu) *FgNEO1* under the control of *pFgNIA1* [47] to characterize its function. Three CPR transformants (*pFgNIA1-FgNEO1*) were identified by PCR screening (S4A and S4B Fig) and verified by qRT-PCR analysis (Fig 8A). Compared to NO_3_^-^ treatment, the relative reduction (RR; %) in colony diameter and the numbers of conidia and perithecia were significant lower in *pFgNIA1-FgNEO1* transformant than in PH-1 under Glu treatment (Fig 8B and 8D). Meanwhile, formation of asci and ascospores showed no obvious differences between PH-1 and *pFgNIA1-FgNEO1* transformant under NO_3_^-^ or Glu treatment (Fig 8E). On the other hand, *pFgNIA1-FgNEO1* transformant almost completely lost its virulence to wheat germs and wheat heads under Glu treatment (Fig 8F). These results suggest that FgNeo1 played important roles in the development and virulence of *F*. *graminearum*, similar to FgPep12 and FgVam7.

### FgNeo1 is essential for ascospore discharge in an ion-dependent manner in *F*. *graminearum*

**Fig 9:**
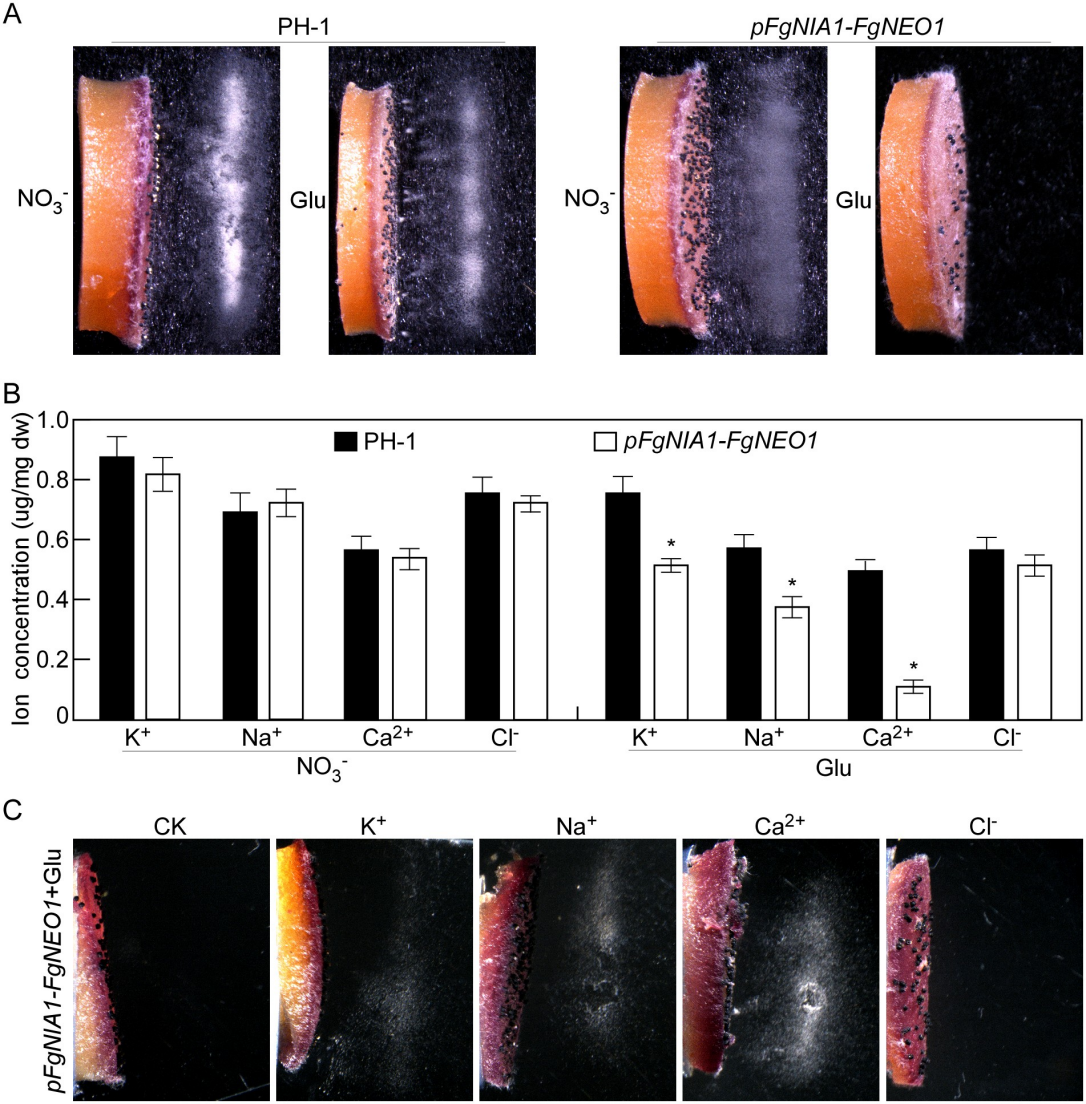
FgNeo1 is essential for ascospore discharge in an ion-dependent manner. (A) Ascospore discharge of perithecia harvested from PH-1 and *pFgNIA1-FgNEO1* cultured on carrot agar plates with NO_3_^-^ or Glu. (B) Measurement of the ion concentration in asci of the indicated strains by spectrophotometrically. Error bars represent the SD and asterisks indicate statistically significant differences (*p*<0.01). (C) Ascospore discharge of perithecia harvested from *pFgNIA1-FgNEO1* cultured on carrot agar plates with Glu by adding exogenous ions.

Although ascus and ascospore formation were not affected by Glu treatment in *pFgNIA1-FgNEO1* transformant, we further investigated whether FgNeo1 plays a role in ascospore discharge. The discharge assay was performed using perithecia from the PH-1 and *pFgNIA1-FgNEO1* strains, treated with NO_3_^-^ and Glu, respectively. No ascospores were discharged from *pFgNIA1-FgNEO1* transformant treated with Glu. In contrast, numerous ascospores were visible from PH-1 treated with NO_3_^-^ and Glu, or from *pFgNIA1-FgNEO1* treated with NO_3_^-^ (Fig 9A), suggesting that FgNeo1 plays an essential role in ascospore discharge in *F*. *graminearum*. To determine the reasons for this difference, we measured the concentrations of ions (Ca^2+^, Na^+^, K^+^ and Cl^-^) in asci, and found that the asci of *pFgNIA1-FgNEO1* transformant treated with Glu exhibited lower concentrations of Ca^2+^, Na^+^ and K^+^, but not Cl^-^. In particular, the Ca^2+^ concentration was decreased sharply in comparison to PH-1 treated with NO_3_^-^ and Glu or to *pFgNIA1-FgNEO1* treated with NO_3_^-^ (Fig 9B). To confirm whether the low ion concentrations cause the defect in ascospore discharge, exogenous ions (Ca^2+^, Na^+^, K^+^ and Cl^-^) were applied to the asci of PH-1 and *pFgNIA1-FgNEO1* transformant treated with Glu. The capacity for ascospore discharge was restored by 43.5% with Na^+^, 42.4% with K^+^ and 70.1% with Ca^2+^, but not improved with Cl^-^ treatment in *pFgNIA1-FgNEO1* transformant (Fig 9C; Table 4). These results demonstrate that FgNeo1 plays an essential role in ascospore discharge in an ion (Ca^2+^, Na^+^, and K^+^)-dependent manner in *F*. *graminearum*.

**Table 4:** Ascospore discharge of the indicated strains under various ions treatment.

Strains	Compounds				
	Water Agar	1 mM NH_4_Cl	1 mM NaCl	1 mM KCl	1 mM CaCl_2_
PH-1+Glu	146.4 ± 5.8	137.5 ± 3.7	138.7 ± 2.9	148.7 ± 4.2	143.7 ± 5.8
*pFgNIA1-FgNEO1*+Glu	0	0	60.4 ± 6.6*	63.1 ± 7.1*	100.7 ± 4.6*

Each number indicates the ascospores discharged from 35 perithecia. ±SD was calculated from three independent experiments, and asterisks indicate statistically significant differences (*p*<0.01).

### FgNeo1 exhibits a late endosome/Golgi localization pattern modulated by FgPep12

**Fig 10:**
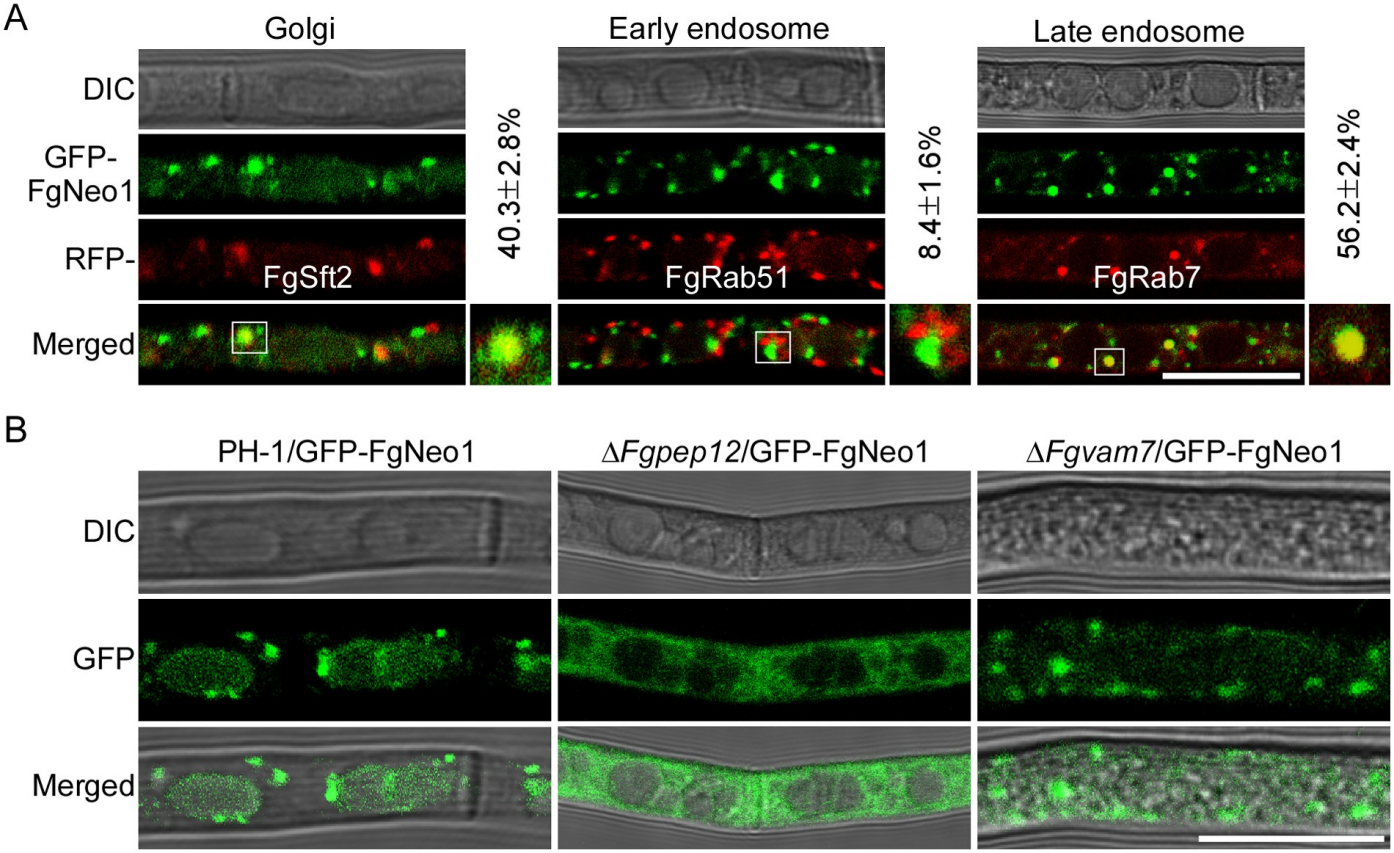
FgNeo1 localizes to late endosomes and Golgi apparatus that regulated by FgPep12. (A) Expression and localization of GFP-FgNeo1 in *F*. *graminearum*. Hyphae of transformants co-expressing GFP-FgNeo1&FgSft2-RFP, GFP-FgNeo1&RFP-FgRab51 or GFP-FgNeo1&RFP-FgRab7 constructs were examined under a confocal microscope. Numbers indicate the merged rate of both proteins. Bar = 10 μm. (B) Hyphae of transformants expressing GFP-FgNeo1 in the wild type PH-1, Δ*Fgpep12* and Δ*Fgvam7* mutants were examined by DIC or fluorescence microscope. Bar = 10 μm.

To investigate how FgNeo1 functions in *F*. *graminearum*, we obtained the GFP-FgNeo1 fluorescence strain and examined its localization pattern in the hyphae. Green fluorescence signals were mainly observed in punctate structures throughout the cell (Fig 10A). In yeast, Neo1 reportedly localizes to the endosomes and Golgi apparatus [48]. Therefore, we co-expressed GFP-FgNeo1 with the Golgi marker protein FgSft2 and the early and late endosome marker proteins FgRab51 and FgRab7 fused to the RFP protein in *F*. *graminearum*. Fluorescence observation revealed that most GFP-FgNeo1 signals overlapped well with RFP-FgSft2 and RFP-FgRab7, but few were co-located with RFP-FgRab51. The percentage of punctate structures exhibiting GFP-FgNeo1 co-localization with RFP-FgSft2 was 40.3%, compared to 56.2% with RFP-FgRab7 and 8.4% with RFP-FgRab51 (Fig 10A), suggesting that GFP-FgNeo1 is mainly localized to the Golgi apparatus and late endosomes in *F*. *graminearum*. We also examined the localization pattern of GFP-FgNeo1 in the Δ*Fgvam7* and Δ*Fgpep12* mutants. Interestingly, GFP-FgNeo1 was mislocalized to the cytosol in the Δ*Fgpep12* mutant, but its localization pattern was not altered notably in the Δ*Fgvam7* mutant (Fig 10B). These findings indicate that FgNeo1 functions within the endomembrane/Golgi system and is closely related to FgPep12.

### Deletion of FgPep12 affects the trafficking of FgVam7&FgNeo1

**Fig 11:**
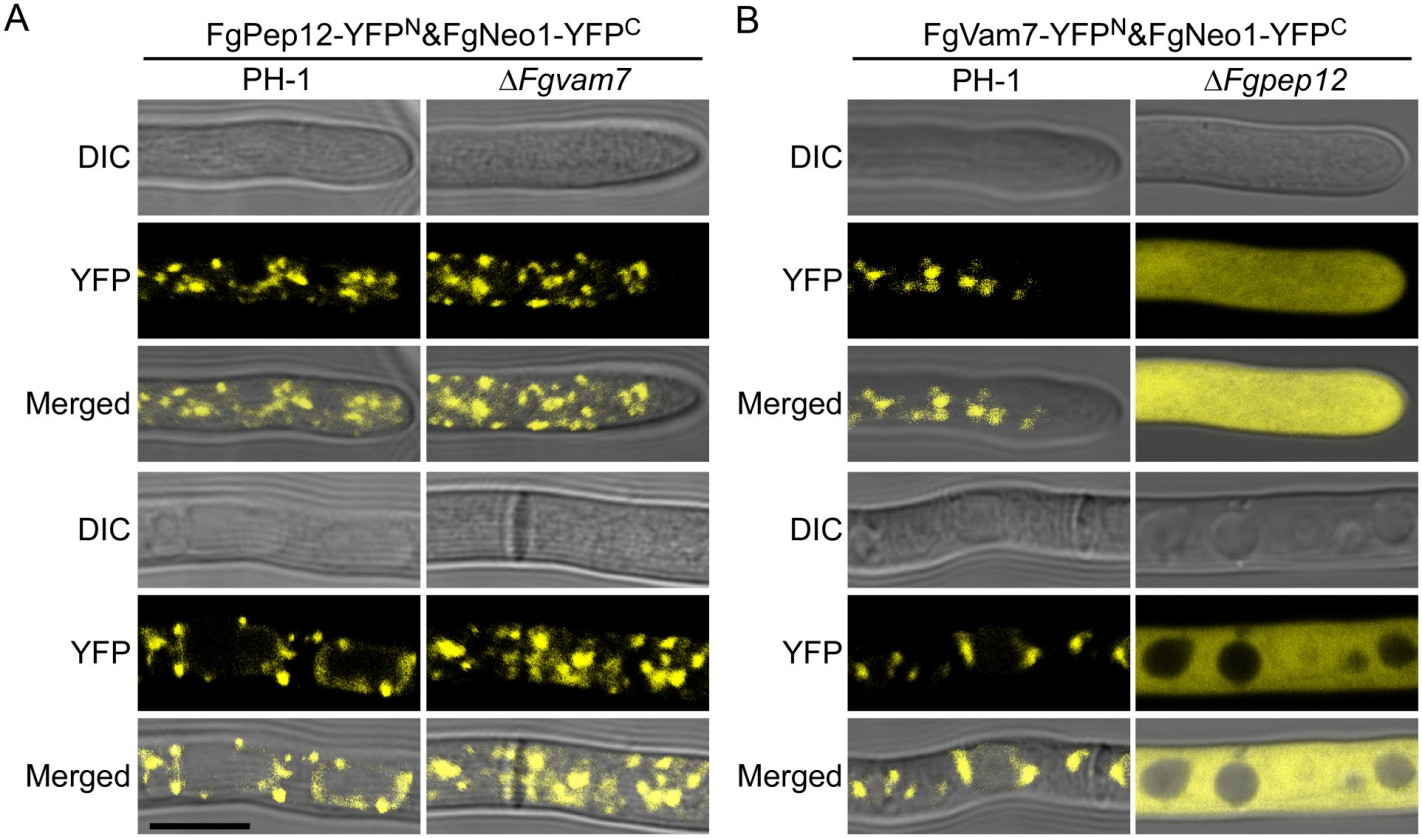
Deletion of FgPep12 affects the localization pattern of FgVam7&FgNeo1. **(A)** Hyphae of transformants expressing FgPep12-YFP^N^&FgNeo1-YFP^C^ in the wild type PH-1 and Δ*Fgvam7* were examined by DIC or fluorescence microscope. (B) Hyphae of transformants expressing FgVam7-YFP^N^&FgNeo1-YFP^C^ in PH-1 and Δ*Fgpep12* were examined by DIC or fluorescence microscope. Bar = 10 μm.

To further clarify the biological significance of interactions among FgPep12, FgVam7 and FgNeo1, we obtained transformants expressing FgPep12-YFP^N^&FgNeo1-YFP^C^ in the Δ*Fgvam7* mutant and FgVam7-YFP^N^&FgNeo1-YFP^C^ in the Δ*Fgpep12* mutant. Microscopic observation revealed that strong yellow fluorescence signals remain visible in both mutants (Fig 11A and 11B), suggesting that deletion of either FgPep12 or FgVam7 does not change the interaction between the other protein and FgNeo1. However, the YFP signal distribution was markedly different in the Δ*Fgpep12* mutant, with a uniform distribution in the cytosol (tip hyphae) or cytosol aside from vacuoles (basal hyphae), in comparison to localization in punctate structures in the wild type and Δ*Fgvam7* mutant (Fig 11A and 11B). Similar results were also observed in ascospores in these two mutants (S5A and S5B Fig). Therefore, deletion of FgPep12 affects the normal transport of FgVam7 and FgNeo1 in *F*. *graminearum*.

## Discussion

**Fig 12:**
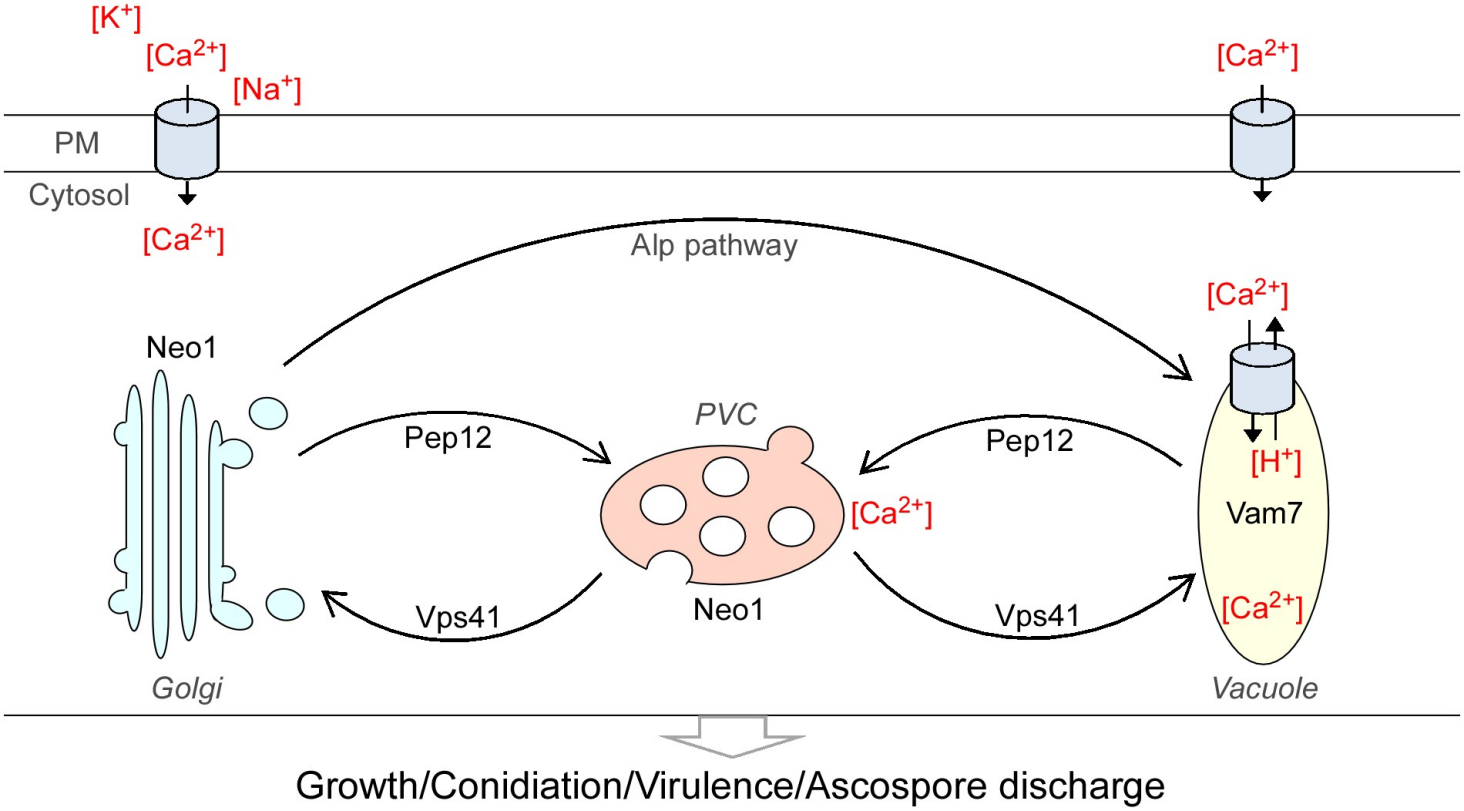
A proposed model for FgPep12-Vam7-Neo1 during the development, plant infection and ascospore discharge in *F*. *graminearum*.

In ascomycetous fungi, the most unique cell type in the life cycle is the ascus. Asci elongate in response to increased turgor pressure and eventually rupture at the tip to fire spores into the air [49]. Under optimal conditions, the asci of *F*. *graminearum* fire individually in succession approximately 45 s apart [29], indicating that some regulatory mechanism must coordinate ascospore discharge. Previous studies have reported that ion channel proteins are closely related to this biological process. Several mutants of ion channel proteins exhibited defects in ascus turgor pressure and failed to discharge ascospores [24, 30–32]. In this study, we identified and characterized the SNARE protein FgPep12, which mediates vacuole-to-PVC transport and is important for the development and virulence of *F*. *graminearum*. Moreover, we unraveled the regulatory mechanisms by which the SNARE proteins FgPep12 and FgVam7 manipulate ascospore discharge in this important plant pathogenic fungus, based on genetic evidence and cytological examination. FgPep12 associated with FgVam7, modulates the trafficking of the Ca^2+^ ATPase FgNeo1 between the Golgi and endosomal/vacuolar system, thereby controlling the vegetative growth, asexual development, ascospore discharge and plant infection processes in *F*. *graminearum* (Fig 12).

SNARE proteins are known to mediate membrane fusion events between transport vesicles and their target membranes in eukaryotic organisms [50–51]. In several plant pathogens, SNARE and associated proteins have been reported to play crucial roles in development and virulence through their involvement in vesicle trafficking pathways. For example, the SNARE protein FgVam7 mediates endocytosis and vacuole membrane fusion, and is important for fungal development and infection [10]. FgVps41, a vacuolar sorting protein associated with FgVam7, mediates membrane fusion and PVC-to-vacuole trafficking. It is also involved in morphogenesis during infectious development [43]. These reports suggested that vesicle trafficking pathways and their components are indispensable for the normal development and virulence of *F*. *graminearum*. The findings of the present study regarding FgPep12 support this conclusion.

Membrane fusion along the secretory pathway requires the specific interaction of SNAREs that are localized to vesicles and target organelles [51]. In SNAREs, the SNARE domain acts as a protein-protein interaction module and is required for SNARE complex formation [52]; the TM domain is anchored to membranes by most SNAREs and has a direct role in fusion [53–54], indicating SNARE and TM domains are important for SNARE functions and for complete fusion. In agree with this, deletion either SNARE or TM in FgPep12 altered its localization pattern and functions. In GFP-ΔSNARE, because of no SNARE domain, FgPep12 probably was unable to interact with other partners that localized to vesicles and target organelles, vacuole fusion might be blocked at an early stage, thus the GFP-ΔSNARE protein failed to accumulate in the vacuoles. In GFP-ΔTM, GFP signals mainly observed in pre-vacuole structures but not mature vacuoles. It seems the formation of SNARE complex was not obviously influenced in GFP-ΔTM, but vacuole fusion was blocked at a late stage because of no TM domain. These findings were also similar to that reported in yeast, in which TM domain performed important roles in the prevacuolar and vacuolar SNARE complexes [55]. In addition, alteration of the TM domain of the SNARE protein Vam3 in yeast affects the composition of the SNARE complex to promote vacuole fusion [54]. TM domain of the SNARE protein Syb2 in neurosecretory cells was reported to play a role in determining early fusion pore structure as well as fusion pore expansion [56]. We speculate that the TM domain of FgPep12 might also have similar functions, but the underlying fusion mechanism needs to be further investigated.

The PVC is a dynamic organelle that receives proteins from a variety of sources, including the biosynthetic and endocytic pathways, as well as the retrograde pathway from vacuoles [40, 57]. Pep12 is involved in all reported membrane fusion events of the PVC in *S*. *cerevisiae* [41]. In this study, we demonstrated that FgPep12 is required for retrograde transport from the vacuole to PVC, and this retrograde transport requires the activity of FgVps41 in *F*. *graminearum*. These results indicate that Pep12 mediates a conserved mechanism in different fungi. The intracellular trafficking systems and related organelles are known to play key roles in the development and pathogenicity of filamentous fungi [5, 39, 58]. For example, retrograde trafficking from the endosome to the trans-Golgi network mediated by the retromer is necessary for development and virulence in *F*. *graminearum* [39]; late endosomal compartments function as important anchors, integrating trafficking and signal transduction of G-proteins and cAMP synthesis during pathogenesis in *M*. *oryzae* [59]; long-distance retrograde motility of early endosomes is essential for *U*. *maydis* effector production and subsequent secretion during plant infection [60]. Combining these reports with our findings, we conclude that FgPep12 is involved in trafficking between the Golgi and endosomal/vacuolar system in *F*. *graminearum*, and may be responsible for protein recycling to maintain fungal development and pathogenesis.

In eukaryotic cells, the calcium ion (Ca^2+^) functions as a ubiquitous intracellular messenger regulating a myriad of biological processes, and the calcium homeostasis system includes tightly regulated metabolic pathways used by cells to maintain Ca^2+^ concentration within the optimal range in the cytosol and other organelles [61–64]. Extracellular Ca^2+^ normally enters the cytosol of cells through various transporters, such as ion channel proteins [65]. In our study, we found that the ascospore discharge defect of Δ*Fgpep12* and Δ*Fgvam7* mutants was closely related to intracellular Ca^2+^ concentrations, indicating that FgVam7 and FgPep12 might have close relationships with calcium signaling and transport pathways. Furthermore, we identified the Ca^2+^ ATPase FgNeo1, which forms a complex with FgVam7 and FgPep12 and is also important for fungal development and ascospore discharge in an ion-dependent manner. Further characterization revealed that FgNeo1 is involved in protein trafficking between the Golgi and endosomal/vacuolar system, which is consistent with findings in yeast [48]. In addition, the localization pattern of FgNeo1 was affected in the Δ*Fgpep12* mutant but not the Δ*Fgvam7* mutant. The reason for this difference might be that FgPep12 showed a similar localization pattern to FgNeo1, and FgPep12 is likely one of most important transporters of FgNeo1 during its trafficking. This possibility was also supported by our BiFC data. FgPep12 and FgNeo1 co-localized in both the Golgi apparatus and late endosomes, while FgVam7 and FgNeo1 mainly co-occurred in late endosomes. In addition, we found that deletion of FgVam7 had no effect on the interaction and localization of FgPep12&FgNeo1, whereas deletion of FgPep12 affected the localization of FgVam7&FgNeo1. These findings indicate that FgPep12 probably plays a major role in the FgPep12-Neo1-Vam7 complex during its trafficking between the Golgi and endosomal/vacuolar system. Mutation of FgPep12 likely blocks the transportation of FgNeo1, thereby disrupting intracellular calcium homeostasis and subsequently affecting the development, plant infection and ascospore discharge processes of *F*. *graminearum*. In regard to FgVam7, PVC-to-vacuole trafficking is constitutively blocked in the Δ*Fgvam7* mutant, as it was defective in vacuole membrane fusion [10]. Thus, cytosolic Ca^2+^ could not be transported into large vacuoles, leading to a defect in calcium homeostasis in cells.

In summary, we identified and characterized the SNARE protein FgPep12 in *F*. *graminearum* and found that FgPep12 is important for fungal development and virulence through its involvement in vesicle trafficking between the Golgi and endosomal/vacuolar system. We further provide multiple lines of evidence showing that SNARE proteins modulate development and ascospore discharge in pathogenic fungi. FgPep12, associated with FgVam7, is required for the trafficking of the Ca^2+^ ATPase FgNeo1 between the Golgi and endosomal/vacuolar system, thus controlling growth, asexual development, ascospore discharge and plant infection in *F*. *graminearum*.

## Materials and methods

### Fungal strains and culture conditions

The *F*. *graminearum* wild type strain PH-1 [66] was used as parental strain for the transformation experiments to gain gene deletion mutants. All strains were cultured on V8 juice agar plates at 25°C in darkness. For vegetative growth assays, all strains were cultured on complete medium (CM) and V8 medium at 25°C for 3 days in darkness. For sporulation, all strains were cultured in liquid carboxymethylcellulose (CMC) medium and assayed as described previously [5, 10, 67]. Mycelia harvested from liquid CM medium were used for genomic DNA and total RNA extractions. Protoplast preparation and fungal transformation were used for PEG-mediated transformation as described previously [68].

### Construction of the gene and domain deletion mutants, and the complemented strains

The *FgPEP12* deletion construct was generated using the split-marker approach [10]. The PCR products were transformed into protoplasts of the wild type PH-1 as described [34, 69]. The resulting transformants were screened by PCR and further confirmed by southern blot analysis. For complementation, the fragment containing the entire *FgPEP12* gene and GFP sequence under the control of *RP27* constitutive promoter, was amplified with primers, and co-transformed with *Xho*I digested pYF11 (neomycin resistance) plasmid using the yeast gap repair approach [68]. The GFP-*FgPEP12* complemented strains were screened by PCR and GFP signal observation. For FgPep12 domain deletion constructs generation, primers were designed according to Splicing Overlap Extension (SOE)-PCR. The PCR products were inserted into pYF11 plasmid and verified by sequencing analysis, and transformed into protoplasts of the Δ*Fgpep12* mutant. The resulting transformants were screened by GFP signals and the final strains were selected for phenotype analysis. The primers used in this section are listed in S1 Table.

### Wheat infection and DON production assays

The infection assay on flowering wheat inflorescences was performed as described previously [5]. A floret of the flowering wheat head was inoculated with 10 μl of conidial suspensions (1×10^5^ conidia/ml). Infected spikelets with typical head blight symptoms were analyzed at two weeks after inoculation. To evaluate the hyphal colonization on infected wheat spikelets, hyphae were examined with a Hitachi TM-1000 tabletop microscope (Hitachi, Tokyo, Japan) at 7 days post-inoculation (dpi). For DON production determination, five mycelial plugs of each strain were inoculated with 50 g healthy and autoclaved wheat kernels [5, 36]. After incubation for 20 d at 25°C, DON was extracted according to a protocol described previously [70]. PuriTox SR DON colummTC-T200 (Trilogy an alytical laboratory) was used to purify the DON, and a HPLC system Waters 1525 (Waters Co., Massachusetts, USA) was used to determine the DON and fungal ergosterol of each sample. The experiment was repeated three times.

### Quantitative RT-PCR analysis

Total RNA samples were extracted using a PureLink TM RNA Mini Kit (Invitrogen) following the instructions. Approximately 1 μg RNA was used for reverse transcription into first strand cDNA using the oligo (dT) primer and M-MLV reverse transcription (Vazyme Biotech, Nanjing, China). Quantitative RT-PCR was performed using an ABI 7500 Fast Real-Time PCR system according to the manufacturer’s instructions. The tubulin beta chain gene (FGSG_09530) was used as the internal control. Relative transcript amount differences were calculated using the 2^-ΔΔCT^ method in ABI 7500 System Sequence Detection Software. Three biological replicates were repeated. The primers used in this section are listed in S1 Table.

### Staining assays

Hyphae were cultured in liquid CM medium for 24 h at 25°C before staining. For vacuole observation, a final concentration of 10 μM of CMAC (7-amino-4-chloromethylcoumarin, Sigma-Aldrich) was used to stain the hyphae or conidia for 30 min at 37°C as described [5, 58, 71]. For conidial septum staining, a final concentration of 10 μg/ml of calcofluor white (CFW) was used in the dark at room temperature for 1 min [72]. Images were taken under Zeiss LSM 710 confocal laser scanning microscope.

### Yeast two hybrid (Y2H), co-immunoprecipitation (co-IP) and bimolecular fluorescence complementation (BiFC) assays

For Y2H assays, the full length cDNA of *FgPEP12*, *FgVAM7* and *FgNEO1* was amplified by PCR with primers and cloned into pGBKT7or pGADT7 as bait or prey constructs. The pairs of prey and bait constructs were co-transformed into the yeast strain AH109 as previously described [73]. The pGBKT7-53 and pGADT7-T were served as positive control, and pGBKT7-Lam and pGADT7-T as negative control. The Trp^+^ and Leu^+^ transformants were isolated and assayed for growth on SD medium stripped of His, Leu and Trp medium. For co-IP assays, the *FgPEP12* and *FgVAM7* gene was cloned into pHZ126 to generate the 3×FLAG fusion constructs, the *FgPEP12* and *FgNEO1* gene was cloned into pYF11 to generate the GFP fusion constructs by yeast gap repair approach. The resulting constructs were verified by sequencing analysis and subsequently co-transformed into the protoplasts of PH-1. Transformants expressing both constructs were screened by GFP signals and confirmed by western blot with anti-FLAG antibody. Total proteins were extracted and incubated with anti-GFP beads as previously described [74]. Proteins eluted from beads were analyzed by western blot with monoclonal anti-FLAG and monoclonal anti-GFP antibodies (Abcam, Cambridge, MA, USA), respectively. The primers used in this section are listed in S1 Table. For BiFC assays, the *FgPEP12* and *FgVAM7* gene was cloned into pHZ65 to generate the YFP^N^ fusion constructs, and the *FgNEO1* gene was cloned into pHZ68 to generate the YFP^C^ fusion construct, by yeast gap repair approach. The resulting constructs were verified by sequencing analysis and co-transformed into the protoplasts of PH-1. The resulting transformants were screened by YFP signals under a fluorescence microscope.

### Ascospore discharge assays

For ascospore discharge, agar blocks covered with perithecia were placed on the end of a glass slide and oriented, and the perithecia bearing surface was perpendicular to the surface of the slide. Then slides were placed on a platform in a transparent humidity chamber under 12 h darkness and 12 h light. Discharged spores were collected from the slides by washing each slide with 1 ml distilled water, and quantified using a hemocytometer.

### Ion channel inhibition assays

Ascospore discharge assays were set up from cultures containing perithecia10 dpi with the following modifications: thickness of the agar blocks was reduced to approximately 2 mm and blocks were placed on a similarly sized 2% water agar block containing dissolved inhibitors. Spores were allowed to discharge from the stacked blocks for 24 h, and then collected and quantified as described above. The number of perithecia on each agar block was also recorded and data normalized to reflect numbers of ascospores discharged per perithecium.

### Statistical analysis

Each result was presented as the mean ± standard deviation (SD) of at least three replicated measurements. The significant differences between treatments were statistically evaluated by SD and one-way analysis of variance (ANOVA) using SPSS 2.0. The data between two specific different treatments were compared statistically by ANOVA, followed by F-test if the ANOVA result is significant at *p*< 0.01.

## Supporting information

S1 Fig Yeast complementation assay. FgPep12 could rescue the growth defect of Δ*Scpep12* mutant under high temperature. Serial dilutions of BY4741, Δ*Scpep12* and Δ*Scpep12* transformed with pYES2 or pYES2-*FgPEP12* were grown on YPD plates at 25°C or 38°C for 2 days and then photographed.
(TIF)

S2 Fig Targeted gene replacement of *FgPEP12* in *F*. *graminearum*. (A) Schematic diagram of deletion strategy of *FgPEP12*. (B) Southern blot analysis of the mutant with gene probe and *HPH* probe, respectively.
(TIF)

S3 Fig FgPep12&FgNeo1 and FgVam7&FgNeo1 mainly localize to Golgi organelles and late endosomes in ascospores. (A and B) Ascospores of transformants expressing RFP-FgSft2 or RFP-FgRab7 constructs in the FgPep12&FgNeo1 and FgVam7&FgNeo1 BiFC strains, were examined under a confocal microscope. Bar = 10 μm.
(TIF)

S4 Fig Targeted replacement of the *FgNEO1* promoter with *pFgNIA1* in *F*. *graminearum*. (A) Strategy for the construction of *pFgNIA1-FgNEO1* CPR transformants. (B) Verification of the candidate CPR transformants (T1, T2, T3) by PCR. +, positive control; -, negative control; CK, using wild type genomic DNA as template.
(TIF)

S5 Fig Deletion of FgPep12 affects the localization of FgVam7&FgNeo1 in ascospores. (A) Ascospores of transformants expressing FgPep12-YFP^N^&FgNeo1-YFP^C^ in the wild type PH-1 and Δ*Fgvam7* were examined by DIC or fluorescence microscope. (B) Ascospores of transformants expressing FgVam7-YFP^N^&FgNeo1-YFP^C^ in PH-1 and Δ*Fgpep12* were examined by DIC or fluorescence microscope. Bar = 10 μm.
(TIF)

S1 Video Dynamics of FgPep12-YFP^N^&FgNeo1-YFP^C^ and its co-localization with RFP-FgRab7. (ZIP)

S2 Video Dynamics of FgPep12-YFP^N^&FgNeo1-YFP^C^ and its co-localization with RFP-FgSft2. (ZIP)

S3 Video Dynamics of FgVam7-YFP^N^&FgNeo1-YFP^C^ and its co-localization with RFP-FgRab7. (ZIP)

S4 Video Dynamics of FgVam7-YFP^N^&FgNeo1-YFP^C^ and its co-localization with RFP-FgSft2. (ZIP)

S1 Table Primers used in this study. (DOC)

## References

[1] Kienle N, Kloepper TH, Fasshauer D. Phylogeny of the SNARE vesicle fusion machinery yields insights into the conservation of the secretory pathway in fungi. BMC Evol Biol. 2009; 9(1): 19 10.1186/1471-2148-9-19 19166604 PMC2639358

[2] Bonifacino JS, Glick BS. The mechanisms of vesicle budding and fusion. Cell. 2004; 116(2): 153–66. 14744428 10.1016/s0092-8674(03)01079-1

[3] Jahn R, Scheller RH. SNAREs—engines for membrane fusion. Nat Rev Mol Cell Bio. 2006; 7(9): 631–43. 10.1038/nrm2002 16912714

[4] Qi ZQ, Liu MX, Dong YH, Zhu Q, Li LW, Li B, et al The syntaxin protein (MoSyn8) mediates intracellular trafficking to regulate conidiogenesis and pathogenicity of rice blast fungus. New Phytol. 2016; 209(4): 1655–67. 10.1111/nph.13710 26522477

[5] Li B, Liu LP, Li Y, Dong X, Zhang HF, Chen HG, et al The FgVps39-FgVam7-FgSso1 complex mediates vesicle trafficking and is important for the development and virulence of *Fusarium graminearum*. Mol Plant Microbe Interact. 2017; 30(5): 410–22. 10.1094/MPMI-11-16-0242-R 28437167

[6] Dou XY, Wang Q, Qi ZQ, Song WW, Wang W, Guo M, et al MoVam7, a conserved SNARE involved in vacuole assembly, is required for growth, endocytosis, ROS accumulation, and pathogenesis of *Magnaporthe oryzae*. PLoS One. 2011; 6(1): e16439 10.1371/journal.pone.0016439 21283626 PMC3025985

[7] Burri L, Lithgow T. A complete set of SNAREs in yeast. Traffic. 2004; 5(1): 45–52. 14675424 10.1046/j.1600-0854.2003.00151.x

[8] Burri L, Varlamov O, Doege CA, Hofmann K, Beilharz T, Rothman JE, et al A SNARE required for retrograde transport to the endoplasmic reticulum. Proc Nat Acad Sci USA. 2003; 100(17): 9873–7. 10.1073/pnas.1734000100 12893879 PMC187870

[9] Song WW, Dou XY, Qi ZQ, Wang Q, Zhang X, Zhang HF, et al R-SNARE homolog MoSec22 is required for conidiogenesis, cell wall integrity, and pathogenesis of *Magnaporthe oryzae*. PLoS One. 2010; 5(10): e13193 10.1371/journal.pone.0013193 20949084 PMC2950850

[10] Zhang H, Li B, Fang Q, Li Y, Zheng X, Zhang Z. SNARE protein FgVam7 controls growth, asexual and sexual development, and plant infection in *Fusarium graminearum*. Mol Plant Pathol. 2016; 17(1): 108–19. 10.1111/mpp.12267 25880818 PMC6638462

[11] Misura KMS, Gonzalez LC, May AP, Scheller RH, Weis WI. Crystal structure and biophysical properties of a complex between the N-terminal SNARE region of SNAP25 and syntaxin 1a. J Bioll Chem. 2001; 276(44): 41301–9. 10.1074/jbc.M106853200 11533035

[12] Pratelli J, Sutter JU, Blatt MR. A new catch in the SNARE. Trends Plant Sci. 2004; 9(4): 187–95. 10.1016/j.tplants.2004.02.007 15063869

[13] Sollner T, Whitehart SW, Brunner M, Erdjumentbromage H, Geromanos S, Tempst P, et al Snap receptors implicated in vesicle targeting and fusion. Nature. 1993; 362(6418): 318–24. 10.1038/362318a0 8455717

[14] Sanderfoot AA, Assaad FF, Raikhel NV. The Arabidopsis genome. An abundance of soluble N-ethylmaleimide-sensitive factor adaptor protein receptors. Plant Physiol. 2000; 124(4): 1558–69. 11115874 10.1104/pp.124.4.1558 PMC59855

[15] Pelham HRB. SNAREs and the secretory pathway—Lessons from yeast. Exp Cell Res. 1999; 247(1): 1–8. 10.1006/excr.1998.4356 10047442

[16] Kuratsu M, Taura A, Shoji J, Kikuchi S, Arioka M, Kitamoto K. Systematic analysis of SNARE localization in the filamentous fungus *Aspergillus oryzae*. Fungal Genet Biol. 2007; 44(12): 1310–23. 10.1016/j.fgb.2007.04.012 17590362

[17] Zuo YS, Yang J, Wang DW, He D, Chu Y, Chen XL, et al MoTlg2, a t-SNARE component is important for formation of the Spitzenkorper and polar deposition of chitin in *Magnaporthe oryzae*. Physiol Mol Plant P. 2014; 87: 9–18.

[18] Hong SY, So J, Lee J, Min K, Son H, Park C, et al Functional analyses of two syntaxin-like SNARE genes, *GzSYN1* and *GzSYN2*, in the ascomycete *Gibberella zeae*. Fungal Genet Biol. 2010; 47(4): 364–72. 10.1016/j.fgb.2010.01.005 20102747

[19] Wedlich-Soldner R, Bolker M, Kahmann R, Steinberg G. A putative endosomal t-SNARE links exo- and endocytosis in the phytopathogenic fungus *Ustilago maydis*. EMBO J. 2000; 19(9): 1974–86. 10.1093/emboj/19.9.1974 10790364 PMC305698

[20] Giraldo MC, Dagdas YF, Gupta YK, Mentlak TA, Yi M, Martinez-Rocha AL, et al Two distinct secretion systems facilitate tissue invasion by the rice blast fungus *Magnaporthe oryzae*. Nat Commun. 2013; 4: 1996 10.1038/ncomms2996 23774898 PMC3709508

[21] Fernando WGD, Paulitz TC, Seaman WL, Dutilleul P, Miller JD. Head blight gradients caused by *Gibberella zeae* from area sources of inoculum in wheat field plots. Phytopathology. 1997; 87(4): 414–21. 10.1094/PHYTO.1997.87.4.414 18945120

[22] Trail F, Xu HX, Loranger R, Gadoury D. Physiological and environmental aspects of ascospore discharge in *Gibberella zeae* (anamorph *Fusarium graminearum*). Mycologia. 2002; 94(2): 181–9. 21156487

[23] Parry DW, Jenkinson P, Mcleod L. Fusarium ear blight (scab) in small-grain cereals-a Review. Plant Pathol. 1995; 44(2): 207–38.

[24] Min K, Lee J, Kim JC, Kim SG, Kim YH, Vogel S, et al A novel gene, *ROA*, is required for normal morphogenesis and discharge of ascospores in *Gibberella zeae*. Eukaryot Cell. 2010; 9(10): 1495–503. 10.1128/EC.00083-10 20802018 PMC2950417

[25] Bai GH, Shaner G. Management and resistance in wheat and barley to Fusarium head blight. Annu Rev Phytopathol. 2004; 42: 135–61. 10.1146/annurev.phyto.42.040803.140340 15283663

[26] Goswami RS, Kistler HC. Heading for disaster: *Fusarium graminearum* on cereal crops. Mol Plant Pathol. 2004; 5(6): 515–25. 10.1111/j.1364-3703.2004.00252.x 20565626

[27] Pestka JJ, Smolinski AT. Deoxynivalenol: Toxicology and potential effects on humans. J Toxicol Env Heal B. 2005; 8(1): 39–69. 10.1080/10937400590889458 15762554

[28] Trail F, Common R. Perithecial development by *Gibberella zeae*: a light microscopy study. Mycologia. 2000; 92(1): 130–8.

[29] Trail F, Gaffoor I, Vogel S. Ejection mechanics and trajectory of the ascospores of *Gibberella zeae* (anamorph *Fuarium graminearum*). Fungal Genet Biol. 2005; 42(6): 528–33. 10.1016/j.fgb.2005.03.008 15878295

[30] Cavinder B, Hamam A, Lew RR, Trail F. Mid1, a mechanosensitive calcium ion channel, affects growth, development, and ascospore discharge in the filamentous fungus *Gibberella zeae*. Eukaryot Cell. 2011; 10(6): 832–41. 10.1128/EC.00235-10 21357477 PMC3127676

[31] Hallen HE, Trail F. The L-type calcium ion channel Cch1 affects ascospore discharge and mycelial growth in the filamentous fungus *Gibberella zeae* (anamorph *Fusarium graminearum*). Eukaryot Cell. 2008; 7(2): 415–24. 10.1128/EC.00248-07 18083828 PMC2238158

[32] Cavinder B, Trail F. Role of Fig1, a component of the low-affinity calcium uptake system, in growth and sexual development of filamentous fungi. Eukaryot Cell. 2012; 11(8): 978–88. 10.1128/EC.00007-12 22635922 PMC3416067

[33] Desjardins AE, Proctor RH, Bai GH, McCormick SP, Shaner G, Buechley G, et al Reduced virulence of trichothecene-nonproducing mutants of *Gibberella zeae* in wheat field tests. Mol Plant Microbe Interact. 1996; 9(9): 775–81.

[34] Proctor RH, Hohn TM, McCormick SP. Reduced virulence of *Gibberella zeae* caused by disruption of a trichothecene toxin biosynthetic gene. Mol Plant Microbe Interact. 1995; 8(4): 593–601. 8589414 10.1094/mpmi-8-0593

[35] Zheng H, Zheng W, Wu C, Yang J, Xi Y, Xie Q, et al Rab GTPases are essential for membrane trafficking-dependent growth and pathogenicity in *Fusarium graminearum*. Environ Microbiol. 2015; 17(11): 4580–99. 10.1111/1462-2920.12982 26177389

[36] Yun YZ, Liu ZY, Zhang JZ, Shim WB, Chen Y, Ma ZH. The MAPKK FgMkk1 of *Fusarium graminearum* regulates vegetative differentiation, multiple stress response, and virulence via the cell wall integrity and high-osmolarity glycerol signaling pathways. Environ Microbiol. 2014; 16(7): 2023–37. 10.1111/1462-2920.12334 24237706

[37] Seong KY, Pasquali M, Zhou X, Song J, Hilburn K, McCormick S, et al Global gene regulation by Fusarium transcription factors Tri6 and Tri10 reveals adaptations for toxin biosynthesis. Mol Microbiol. 2009; 72(2): 354–67. 10.1111/j.1365-2958.2009.06649.x 19320833

[38] Liu YW, Huang CF, Huang KB, Lee FJS. Role for Gcs1p in regulation of Arl1p at trans-Golgi compartments. Mol Biol Cell. 2005; 16(9): 4024–33. 10.1091/mbc.E05-01-0023 15975906 PMC1196316

[39] Zheng WH, Zheng HW, Zhao X, Zhang Y, Xie QR, Lin XL, et al Retrograde trafficking from the endosome to the trans-Golgi network mediated by the retromer is required for fungal development and pathogenicity in *Fusarium graminearum*. New Phytol. 2016; 210(4): 1327–43. 10.1111/nph.13867 26875543

[40] Bryant NJ, Piper RC, Weisman LS, Stevens TH. Retrograde traffic out of the yeast vacuole to the TGN occurs via the prevacuolar/endosomal compartment. J Cell Biol. 1998; 142(3): 651–63. 9700156 10.1083/jcb.142.3.651 PMC2148167

[41] Gerrard SR, Levi BP, Stevens TH. Pep12p is a multifunctional yeast syntaxin that controls entry of biosynthetic, endocytic and retrograde traffic into the prevacuolar compartment. Traffic. 2000; 1(3): 259–69. 11208109 10.1034/j.1600-0854.2000.010308.x

[42] Bryant NJ, Stevens TH. Two separate signals act independently to localize a yeast late Golgi membrane protein through a combination of retrieval and retention. J Cell Biol. 1997; 136(2): 287–97. 9015300 10.1083/jcb.136.2.287 PMC2134822

[43] Li B, Dong X, Li XR, Chen HG, Zhang HF, Zheng XB, et al A subunit of the HOPS endocytic tethering complex, FgVps41, is important for fungal development and plant infection in *Fusarium graminearum*. Environ Microbiol. 2018; 20(4): 1436–51. 10.1111/1462-2920.14050 29411478

[44] Nothwehr SF, Roberts CJ, Stevens TH. Membrane-protein retention in the yeast Golgi-apparatus-dipeptidyl aminopeptidase-a is retained by a cytoplasmic signal containing aromatic residues. J Cell Biol. 1993; 121(6): 1197–209. 8509444 10.1083/jcb.121.6.1197 PMC2119699

[45] Nguyen QB, Kadotani N, Kasahara S, Tosa Y, Mayama S, Nakayashiki H. Systematic functional analysis of calcium-signalling proteins in the genome of the rice-blast fungus, *Magnaporthe oryzae*, using a high-throughput RNA-silencing system. Mol Microbiol. 2008; 68(6): 1348–65. 10.1111/j.1365-2958.2008.06242.x 18433453

[46] Hua Z, Graham TR. Requirement for neo1p in retrograde transport from the Golgi complex to the endoplasmic reticulum. Mol Biol Cell. 2003; 14(12): 4971–83. 10.1091/mbc.E03-07-0463 12960419 PMC284799

[47] Marchegiani E, Sidhu Y, Haynes K, Lebrun MH. Conditional gene expression and promoter replacement in *Zymoseptoria tritici* using fungal nitrate reductase promoters. Fungal Genet Biol. 2015; 79: 174–9. 10.1016/j.fgb.2015.04.021 26092804

[48] Wicky S, Schwarz H, Singer-Kruger B. Molecular interactions of yeast Neo1p, an essential member of the Drs2 family of aminophospholipid translocases, and its role in membrane trafficking within the endomembrane system. Mol Cell Biol. 2004; 24(17): 7402–18. 10.1128/MCB.24.17.7402-7418.2004 15314152 PMC507011

[49] Trail F. Fungal cannons: explosive spore discharge in the Ascomycota. FEMS Microbiol Lett. 2007; 276(1): 12–8. 10.1111/j.1574-6968.2007.00900.x 17784861

[50] Ferro-Novick S, Jahn R. Vesicle fusion from yeast to man. Nature. 1994; 370(6486): 191–3. 10.1038/370191a0 8028665

[51] Rothman JE. Mechanisms of intracellular protein transport. Nature. 1994; 372(6501): 55–63. 10.1038/372055a0 7969419

[52] Katz L, Hanson PI, Heuser JE, Brennwald P. Genetic and morphological analyses reveal a critical interaction between the C-termini of two SNARE proteins and a parallel four helical arrangement for the exocytic SNARE complex. EMBO J. 1998; 17(21): 6200–9. 10.1093/emboj/17.21.6200 9799229 PMC1170946

[53] Weimbs T, Low SH, Chapin SJ, Mostov KE, Bucher P, Hofmann K. A conserved domain is present in different families of vesicular fusion proteins: A new superfamily. Proc Natl Acad Sci USA. 1997; 94(7): 3046–51. 10.1073/pnas.94.7.3046 9096343 PMC20319

[54] Rohde J, Dietrich L, Langosch D, Ungermann C. The transmembrane domain of Vam3 affects the composition of cis- and trans-SNARE complexes to promote homotypic vacuole fusion. J Biol Chem. 2003; 278(3): 1656–62. 10.1074/jbc.M209522200 12427733

[55] Gerrard SR, Mecklem AB, Stevens TH. The yeast endosomal t-SNARE, pep12p, functions in the absence of its transmembrane domain. Traffic. 2000; 1(1): 45–55. 11208059 10.1034/j.1600-0854.2000.010108.x

[56] Fang QH, Lindau M. How could SNARE proteins open a fusion pore? Physiology. 2014; 29(4): 278–85. 10.1152/physiol.00026.2013 24985331 PMC4103061

[57] Bryant NJ, Stevens TH. Vacuole biogenesis in *Saccharomyces cerevisiae*: Protein transport pathways to the yeast vacuole. Microbiol Mol Biol R. 1998; 62(1): 230–47. 9529893 10.1128/mmbr.62.1.230-247.1998 PMC98912

[58] Shoji JY, Arioka M, Kitamoto K. Vacuolar membrane dynamics in the filamentous fungus *Aspergillus oryzae*. Eukaryot cell. 2006; 5(2): 411–21. 10.1128/EC.5.2.411-421.2006 16467481 PMC1405889

[59] Ramanujam R, Calvert ME, Selvaraj P, Naqvi NI. The late endosomal HOPS complex anchors active G-protein signaling essential for pathogenesis in *Magnaporthe oryzae*. PLoS Pathog. 2013; 9(8): e1003527 10.1371/journal.ppat.1003527 23935502 PMC3731250

[60] Bielska E, Higuchi Y, Schuster M, Steinberg N, Kilaru S, Talbot NJ, et al Long-distance endosome trafficking drives fungal effector production during plant infection. Nat Commu. 2014; 5: 5097 10.1038/ncomms6097 25283249 PMC4205857

[61] Berridge MJ, Lipp P, Bootman MD. The versatility and universality of calcium signalling. Nat Rev Mol Cell Bio. 2000; 1(1): 11–21. 10.1038/35036035 11413485

[62] Berridge MJ, Bootman MD, Roderick HL. Calcium signalling: Dynamics, homeostasis and remodelling. Nat Rev Mol Cell Bio. 2003;4(7):517–29. 10.1038/nrm1155 12838335

[63] Cui JJ, Kaandorp JA, Sloot PMA, Lloyd CM, Filatov MV. Calcium homeostasis and signaling in yeast cells and cardiac myocytes. FEMS Yeast Res. 2009; 9(8): 1137–47. 10.1111/j.1567-1364.2009.00552.x 19678847

[64] Netikova K, Sychrova H, Zimmermannova O. Potassium homeostasis and calcium signalling in yeast cells. FEBS Open Bio. 2018; 9(8): 374–5.

[65] Locke EG, Bonilla M, Liang L, Takita Y, Cunningham KW. A homolog of voltage-gated Ca^2+^ channels stimulated by depletion of secretory Ca^2+^ in yeast. Mol Cell Biol. 2000; 20(18): 6686–94. 10958666 10.1128/mcb.20.18.6686-6694.2000 PMC86178

[66] Cuomo CA, Gueldener U, Xu JR, Trail F, Turgeon BG, Di Pietro A, et al The *Fusarium graminearum* genome reveals a link between localized polymorphism and pathogen specialization. Science. 2007; 317(5843): 1400–2. 10.1126/science.1143708 17823352

[67] Zheng DW, Zhang SJ, Zhou XY, Wang CF, Xiang P, Zheng Q, et al The *FgHOG1* pathway regulates hyphal growth, stress responses, and plant infection in *Fusarium graminearum*. PLoS One. 2012; 7(11): e4949 10.1371/journal.pone.0049495 23166686 PMC3498113

[68] Zhou X, Li G, Xu JR. Efficient approaches for generating GFP fusion and epitope-tagging constructs in filamentous fungi. Methods Mol Biol. 2011; 722: 199–212. 10.1007/978-1-61779-040-9_15 21590423

[69] Hou ZM, Xue CY, Peng YL, Katan T, Kistler HC, Xu JR. A mitogen-activated protein kinase gene (*MGV1*) in *Fusarium graminearum* is required for female fertility, heterokaryon formation, and plant infection. Mol Plant Microbe Interact. 2002; 15(11): 1119–27. 10.1094/MPMI.2002.15.11.1119 12423017

[70] Mirocha CJ, Kolaczkowski E, Xie WP, Yu H, Jelen H. Analysis of deoxynivalenol and its derivatives (batch and single kernel) using gas chromatography mass spectrometry. J Agr Food Chem. 1998; 46(4): 1414–8.

[71] Ohneda M, Arioka M, Nakajima H, Kitamoto K. Visualization of vacuoles in *Aspergillus oryzae* by expression of CPY-EGFP. Fungal Genet Biol. 2002; 37(1): 29–38. 12223187 10.1016/s1087-1845(02)00033-6

[72] Harris SD, Morrell JL, Hamer JE. Identification and characterization of *Aspergillus nidulans* mutants defective in cytokinesis. Genetics. 1994; 136(2): 517–32. 8150280 10.1093/genetics/136.2.517 PMC1205805

[73] Schiestl RH, Gietz RD. High-efficiency transformation of intact yeast-cells using single stranded nucleic-acids as a carrier. Curr Genet. 1989; 16(5–6): 339–46. 2692852 10.1007/BF00340712

[74] Li LW, Chen XL, Zhang SP, Yang J, Chen D, Liu MX, et al MoCAP proteins regulated by MoArk1-mediated phosphorylation coordinate endocytosis and actin dynamics to govern development and virulence of *Magnaporthe oryzae*. PLoS Genet. 2017;13(5): e100681 10.1371/journal.pgen.1006814 28542408 PMC5466339

