# Supplementary figures and images for: The t-SNARE protein FgPep12, associated with FgVam7, is essential for ascospore discharge and plant infection by trafficking Ca2+ ATPase FgNeo1 between Golgi and endosome/vacuole in Fusarium graminearum
Source: PLoS Pathog. 2019 May 8;15(5):e1007754. doi: 10.1371/journal.ppat.1007754 (PMC6527245; doi:10.1371/journal.ppat.1007754)

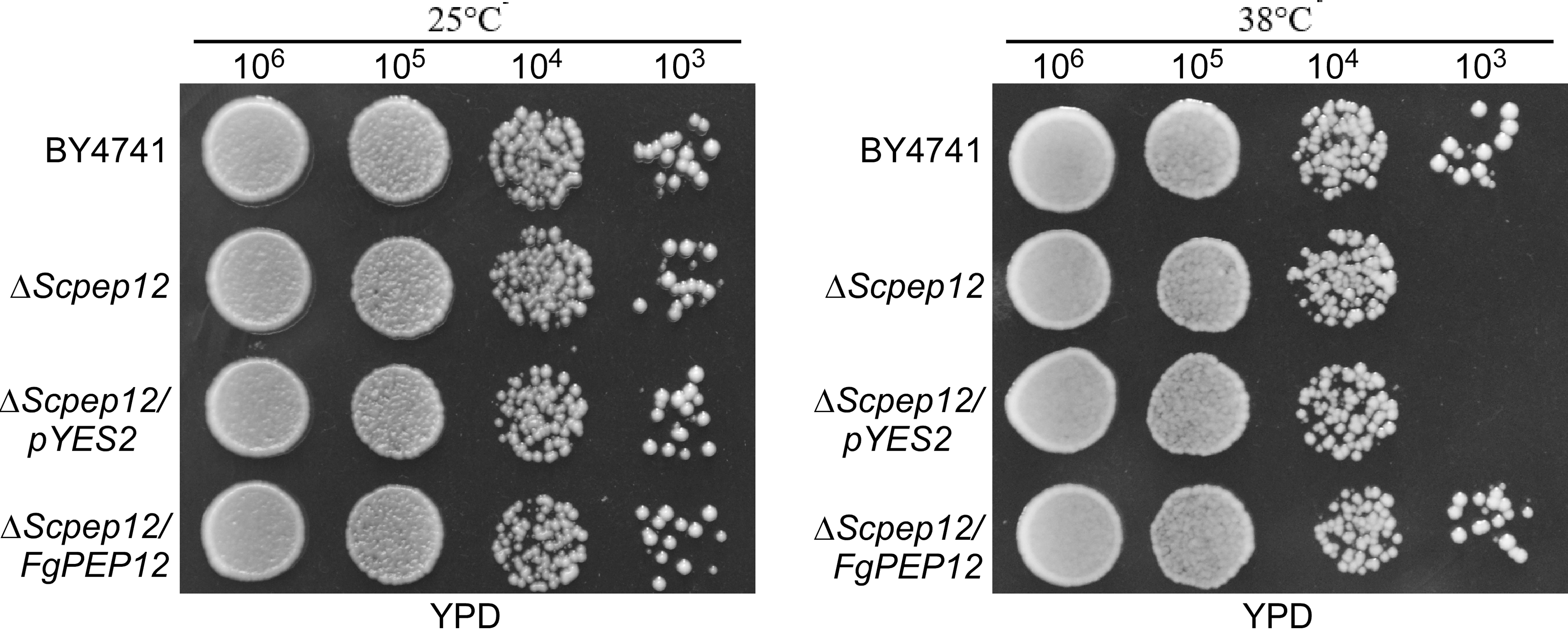

Supplement: S1 Fig — FgPep12 could rescue the growth defect of ΔScpep12 mutant under high temperature. Serial dilutions of BY4741, ΔScpep12 and ΔScpep12 transformed with pYES2 or pYES2-FgPEP12 were grown on YPD plates at 25°C or 38°C for 2 days and then photographed. (TIF) [file ppat.1007754.s001.tif]

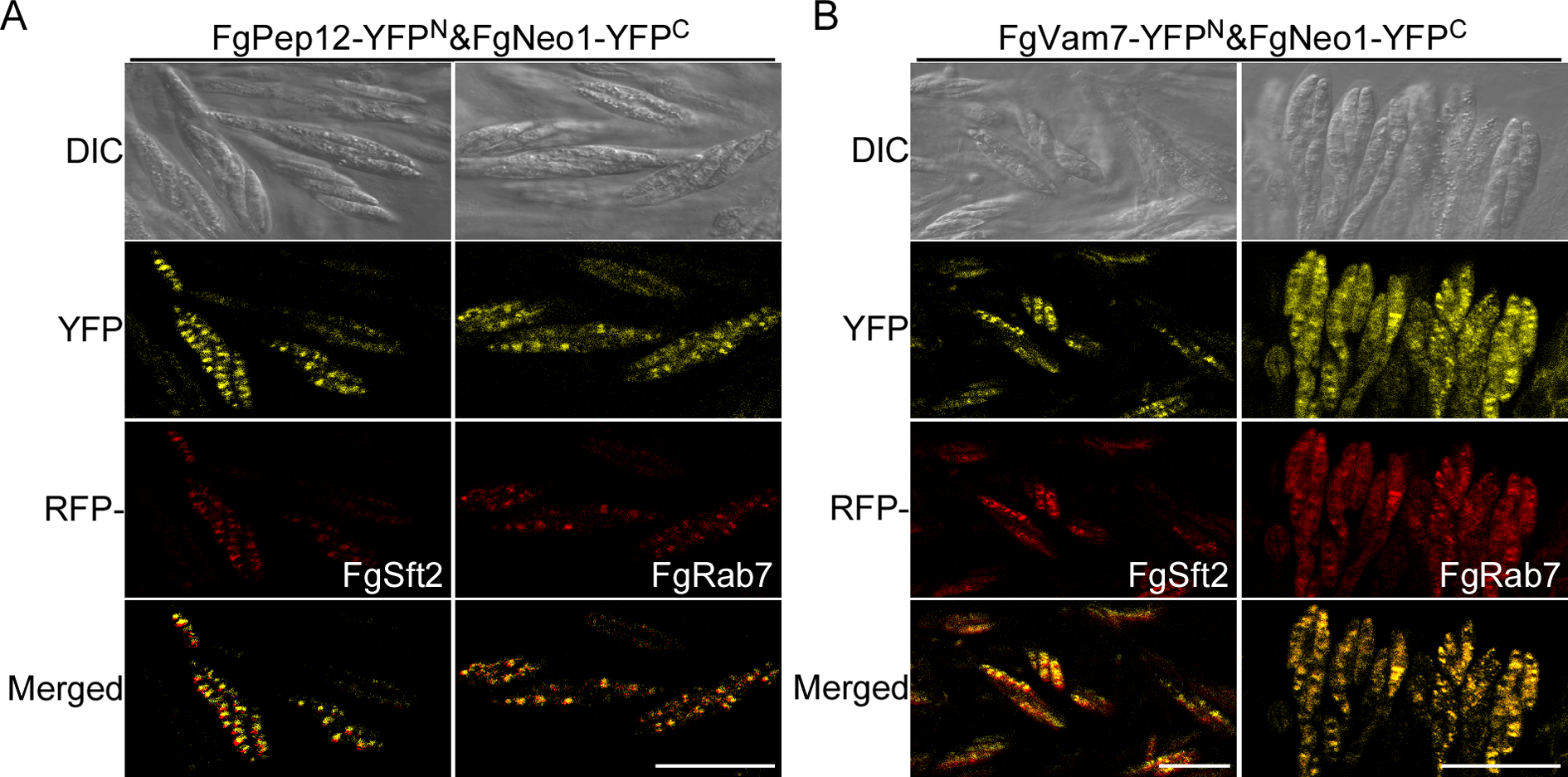

Supplement: S3 Fig — (A and B) Ascospores of transformants expressing RFP-FgSft2 or RFP-FgRab7 constructs in the FgPep12&FgNeo1 and FgVam7&FgNeo1 BiFC strains, were examined under a confocal microscope. Bar = 10 μm. (TIF) [file ppat.1007754.s003.tif]

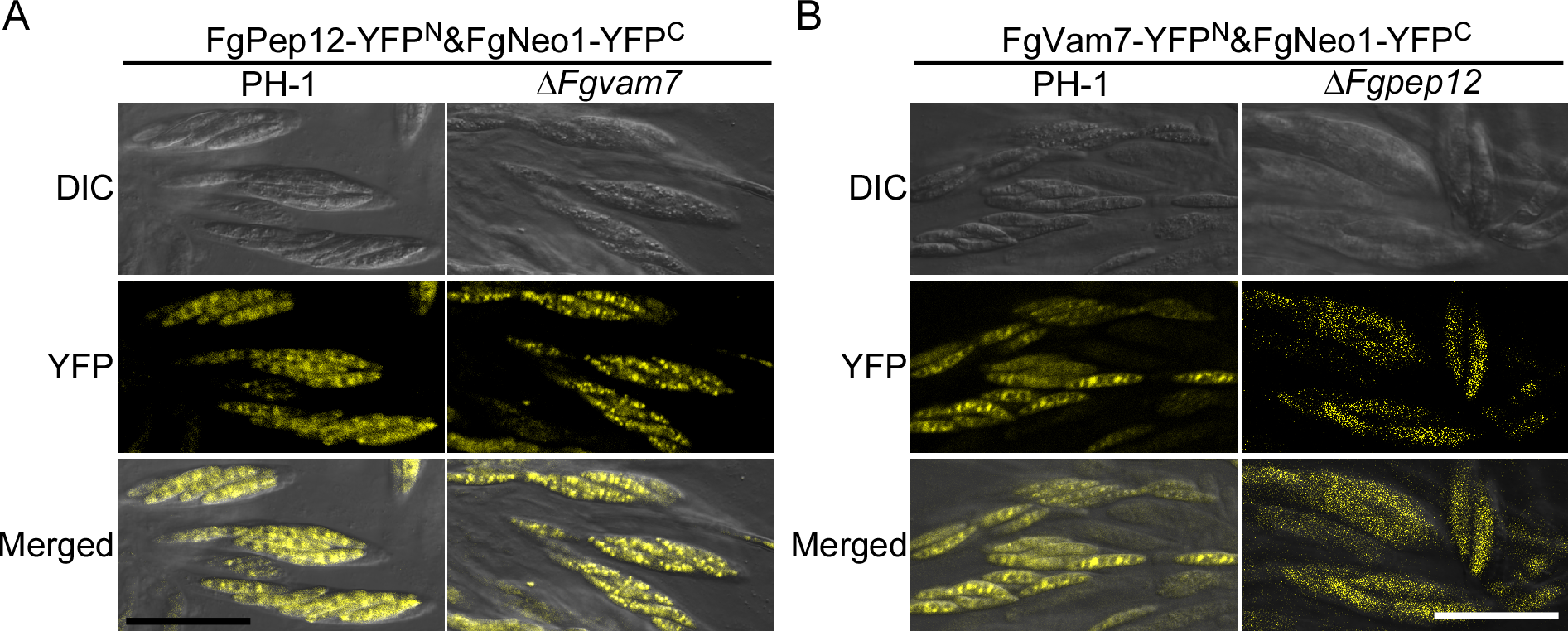

Supplement: S5 Fig — (A) Ascospores of transformants expressing FgPep12-YFPN&FgNeo1-YFPC in the wild type PH-1 and ΔFgvam7 were examined by DIC or fluorescence microscope. (B) Ascospores of transformants expressing FgVam7-YFPN&FgNeo1-YFPC in PH-1 and ΔFgpep12 were examined by DIC or fluorescence microscope. Bar = 10 μm. (TIF) [file ppat.1007754.s005.tif]
