# Supplementary material for: The t-SNARE protein FgPep12, associated with FgVam7, is essential for ascospore discharge and plant infection by trafficking Ca2+ ATPase FgNeo1 between Golgi and endosome/vacuole in Fusarium graminearum
Source: PLoS Pathog. 2019 May 8;15(5):e1007754. doi: 10.1371/journal.ppat.1007754 (PMC6527245; doi:10.1371/journal.ppat.1007754)
Supplement: S1 Table — (DOC) [file ppat.1007754.s010.doc]

**S1 Table. Primers used in this study.**

| **Primer name** | **Oligonucleotide sequence (5’-3’)** | **Remark** |
| --- | --- | --- |
| FgPep12-1F | GTCAGTCCAAGATCCCGAAACA | For *FgPEP12* 5’ flank sequence amplification |
| FgPep12-2R | TTGACCTCCACTAGCTCCAGCCAAGCCGCAAGCGAGTGATCGAATCTATG |
| FgPep12-3F | CAAAGGAATAGAGTAGATGCCGACCGAATCTCGGGAATACCCGCTGTC | For *FgPEP12* 3’ flank sequence amplification |
| FgPep12-4R | GATGGGTCGAGTACCACTCCTT |
| FgPep12-5F | TACCAGTAGCTATCCGAACGAG | For *FgPEP12* gene probe amplification |
| FgPep12-6R | GAACATGTCTGTTGGTAGCTGG |
| FgPep12-7F | AGCAACGACTTCAACTAAGTCG | For transformants screen |
| FgPep12-8R | TGAACTGTACAGTCCGGATCCT |
| HYG/F | GGCTTGGCTGGAGCTAGTGGAGGTCAA | For HPH-N sequence amplification |
| HY/R | GTATTGACCGATTCCTTGCGGTCCGAA |
| YG/F | GATGTAGGAGGGCGTGGATATGTCCT | For HPH-C sequence amplification |
| HYG/R | CGGTCGGCATCTACTCTATTCCTTTG |
| GFP-FgPep12-1F | TTTCGTAGGAACCCAATCTTCAAAATGGTGAGCAAGGGCGAGGAGC | For *GFP-FgPEP12* fusion construct generation |
| GFP-FgPep12-2R | CTTGTACAGCTCGTCCATGCCGAGAGTG |
| GFP-FgPep12-3F | ACTCTCGGCATGGACGAGCTGTACAAGATGTGGCGAGACCGCACCAACCT |
| GFP-FgPep12-4R | CTTCTCGTTGGGGTCTTTGCTCAGGCTACTCGTGTTTCTTGGGTTTGA |
| GFP-1F | TTTCGTAGGAACCCAATCTTCAAAATGGTGAGCAAGGGCGAGGAGC | For *FgPEP12* SNARE domain deletion construct generation |
| GFP-2R | CTTGTACAGCTCGTCCATGCCGAGAGTG |
| FgPep12^ΔSNARE^ -1F | CACTCTCGGCATGGACGAGCTGTACAAGATGTGGCGAGACCGCACCAACC |
| FgPep12^ΔSNARE^-2R | CCTCTGCTGTTGGGTTGCTTGAA |
| FgPep12^ΔSNARE^ -3F | TTCAAGCAACCCAACAGCAGAGGTCAGGCTACCAAAGGAGGAC |
| FgPep12^ΔSNARE^ -4R | CACCACCCCGGTGAACAGCTCCTCGCCCTTGCTCACCTACTCGTGTTTCTTGGGTTTGA |
| GFP-1F | TTTCGTAGGAACCCAATCTTCAAAATGGTGAGCAAGGGCGAGGAGC | For *FgPEP12* TM domain deletion fusion construct generation |
| GFP-2R | CTTGTACAGCTCGTCCATGCCGAGAGTG |
| FgPep12^^ -1F | CACTCTCGGCATGGACGAGCTGTACAAGATGTGGCGAGACCGCACCAACC |
| FgPep12^^ -2R | CACCACCCCGGTGAACAGCTCCTCGCCCTTGCTCACCTACTCGTGTTTCTTGGGTTTCTTGCGTTTCGTCGTCCTCCTT |
| TRI5-QF | TGAGGGATGTTGGATTGAGCA | For *FgTRI5* qRT-PCR analysis |
| TRI5-QR | TGCTTCCGCTCATCAAACAGG |
| TRI6-QF | GCTACTCAGAATGCCCTCAG | For *FgTRI6* qRT-PCR analysis |
| TRI6-QR | CGCATGTTATCCACCCTGCTA |
| RFP-FgSft2-1F | TTTCGTAGGAACCCAATCTTCAAAATGGCCTCCTCCGAGGACGTC | For *RFP-FgSFT2* fusion construct generation |
| RFP-FgSft2-2R | GGCGCCGGTGGAGTGGCGGCCCT |
| RFP-FgSft2-3F | AGGGCCGCCACTCCACCGGCGCCATGGCTTCTTCTTCCTTCCGA |
| RFP-FgSft2-4R | CACCACCCCGGTGAACAGCTCCTCGCCCTTGCTCACCTACCCAGTCATCCAGGCTGTT |
| FgVam7-RFP-1F | TTTCGTAGGAACCCAATCTTCAAAATGACCGCGGCGCATTCGTCACT | For *FgVAM7-RFP* fusion construct generation |
| FgVam7-RFP-2R | ACGTCCTCGGAGGAGGCCATCATCTTCTTAATACGGTTGTTGGC |
| RFP-1F | TTTACAAACGATTATTCACCATGGCCTCCTCCGAGGACGTC | For *RFP-FgRAB7* fusion construct generation |
| RFP-2R | GGCGCCGGTGGAGTGGCGGCCCTCGG |
| RFP-FgRab7-1F | GGGCCGCCACTCCACCGGCGCCATGTCTTCTCGAAAGAAGGTTC |
| RFP-FgRab7-2R | CACCACCCCGGTGAACAGCTCCTCGCCCTTGCTCACTTAACAAGCACAGCCATCGCGGTCGTTCTC |
| FgVam7-flag-1F | CTATAGGGCGAATTGGGTACTCAAATTGGTTGTTGGCTATAGCAGATAGCTAC | For *FgVAM7-FLAG* fusion construct generation |
| FgVam7-flag-2R | CTTTATAATCACCGTCATGGTCTTTGTAGTCCATCTTCTTAATACGGTTGTTGG |
| AD-FgPep12-1F | TAAGAATTCATGTGGCGAGACCGCACCAACCT | For pGADT7*-FgPEP12*  fusion construct generation |
| AD-FgPep12-2R | TAAGGGATCCATCTACTCGTGTTTCTTGGGTTTGA |
| AD-FgVam7-1F | TAACATATGATGACCGCGGCGCATTCCGTATCCGATGAAGGATCAA | For pGADT7*-FgVAM7* fusion construct generation |
| AD-FgVam7-2R | TAAGAATTCTCACATCTTCTTAATACGGTTGTTGGCTACCTTTAGC |
| BD-FgVam7-1F | TAACATATGATGACCGCGGCGCATTCCGTATCCGATGAAGGATCAA | For pGBKT7*-FgVAM7* fusion construct generation |
| BD-FgVam7-1F | TAAGAATTCTCACATCTTCTTAATACGGTTGTTGGCTACCTTTAGC |
| RP27-ScALP-GFP-1F | TTTCGTAGGAACCCAATCTTCAAAATGATGACTCACACATTACCAA | For *ScALP-GFP* fusion construct generation |
| RP27-ScALP-GFP-2R | CACCACCCCGGTGAACAGCTCCTCGCCCTTGCTCACCTTTGATCTCTTCGAGATCCGT |
| RP27-RS-ScALP-GFP-1F | AGCGATCACTACCGGCGTCATCC | For *RS-ScALP-GFP* fusion construct generation |
| RP27-RS-ScALP-GFP-2R | CAAGTCATTGAATTGGAATGATTCGCGGCGCTGTTCGCTTGGTAATGTGTGAGTCA |
| RP27-RS-ScALP-GFP-3F | TGACTCACACATTACCAAGCGAACAGCGCCGCGAATCATTCCAATTCAATGACTTG |
| RP27-RS-ScALP-GFP-4R | ATGATATAGACGTTGTGGCTGTTGTAGTTGT |
| RP27-(F/A)RS-ScALP-GFP-1F | AGCGATCACTACCGGCGTCATCC | For *(F/A)RS-ScALP-GFP* fusion construct generation |
| RP27-(F/A)RS-ScALP-GFP-2R | CAAGTCATTAGCTTGAGCTGATTCGCGGCGGCTTTCGTTGGCCTGAGGAGATGA |
| RP27-(F/A)RS-ScALP-GFP-3F | TCATCTCCTCAGGCCAACGAAAGCCGCCGCGAATCAGCTCAAGCTAATGACTTG |
| RP27-(F/A)RS-ScALP-GFP-4R | ATGATATAGACGTTGTGGCTGTTGTAGTTGT |
| pHZ65- FGSG_05149- 1F | CGACTCACTATAGGGCGAATTGGGTACTCAAATTGCTGATGTGTATTATTGTATCGGTT | For pHZ65*-FGSG_05149*  fusion construct generation |
| pHZ65 -FGSG_05149- 2R | GCTCACCATCGTGGCGATGGAGCGACGATTTTGAACCTTCCTGTAAGA |
| pHZ68-FgPep12-1F | CGACTCACTATAGGGCGAATTGGGTACTCAAATTGAGCTTCTCGCCGATGGAGGTCA | For pHZ68*-FgPEP12* fusion construct generation |
| pHZ68-FgPep12-2R | GTTCGGGATCTTGCAGGCCGGGCGCTCGTGTTTCTTGGGTTTGATCA |
| pHZ68-FgVam7-1F | CGACTCACTATAGGGCGAATTGGGTACTCAAATTGTGCAGCAGGAGAACGCTACACTG | For pHZ68-*FgVAM7* fusion construct generation |
| pHZ68-FgVam7-2R | GTTCGGGATCTTGCAGGCCGGGCGCATCTTCTTAATACGGTTGTTGG |
| BD-FGSG_01196-1F | CTGATCTCAGAGGAGGACCTGATGGCTGACCCCGATCTACCAA | For pGBKT7*-*FGSG*_*01196  fusion construct generation |
| BD-FGSG_01196- 2R | TGCAGGTCGACGGATCCCCGGTTAGGAGTGACCCCCTTCAGT |
| BD-FGSG_07518-1F | CTGATCTCAGAGGAGGACCTGATGGACGACATTACGACTGC | For pGBKT7*-*FGSG*_*07518  fusion construct generation |
| BD-FGSG_07518-2R | TGCAGGTCGACGGATCCCCGGTTATCGCCTCTTAGATCTCAAC |
| BD-FGSG_08758-1F | CTGATCTCAGAGGAGGACCTGATGTCGAGCAAACACGCAAAC | For pGBKT7-FGSG*_*08758  fusion construct generation |
| BD-FGSG_08758-2R | TGCAGGTCGACGGATCCCCGGTTAGTTGCTAGGGTCCAT |
| BD-FGSG_09515-1F | CTGATCTCAGAGGAGGACCTGATGGCTGATCATGATGAC | For pGBKT7-FGSG*_*09515  fusion construct generation |
| BD-FGSG_09515-2R | TGCAGGTCGACGGATCCCCGGCTACTTTTGTGCATTTGTGTTGTCTCC |
| BD-FGSG_01265-1F | CTGATCTCAGAGGAGGACCTGATGGAGACCGCCTTCGCGAAGC | For pGBKT7*-FGSG_*01265  fusion construct generation |
| BD-FGSG_01265-2R | TGCAGGTCGACGGATCCCCGGCTATTGCTCTTTCTTTGTCTT |
| BD-FGSG_04178-1F | CTGATCTCAGAGGAGGACCTGATGTCTGGCACCTGGACAC | For pGBKT7-FGSG_04178  fusion construct generation |
| BD-FGSG_04178-2R | TGCAGGTCGACGGATCCCCGGCTAATTGCTAGAGCCATCCT |
| BD-FGSG_05149-1F | CTGATCTCAGAGGAGGACCTGATGCCGCCCTCACAACAAT | For pGBKT7-FGSG_05149  fusion construct generation |
| BD-FGSG_05149-2R | TGCAGGTCGACGGATCCCCGGTTAACGATTTTGAACCTTCCTG |
| BD-FGSG_13640-1F | CTGATCTCAGAGGAGGACCTGATGTCACGAAATGGTACCAC | For pGBKT7-FGSG*_*13640  fusion construct generation |
| BD-FGSG_13640-2R | TGCAGGTCGACGGATCCCCGGTCATCGTCCTGTCAAACTGGAT |
| FGSG_05149-1F | TCCTTCGTCTCTCGGACCTTG | For *pFgNEO1* 5’ flank sequence amplification |
| FGSG_05149-2R | CATTGATGTGTTGACCTCCCAAGGTCCGAGAGACGAAGGA |
| FGSG_05149-3F | ATGCCGCCCTCACAACAATAC | For *pFgNEO1* 3’ flank sequence amplification |
| FGSG_05149-4R | AGTCCTTGGACTTGCGGCTG |
| FGSG_05149-5F | CGGCTTAATCCAACTTGCCG | Validation of *pFgNEO1* deletion |
| FGSG_05149-6R | CGTGTCGCATTTATTCCAGG |
| FGSG_05149-7F | GCGAAGCTTCTTTTGTCC |
| FGSG_05149-8R | GCAGGTTCCTCGGGAATAGG |
| HCR | GATGCTTGGGTAGAATAGG |
| pNF | GCGAAGAGAGCACCTTGGTTTA | Amplification of p*FgNIA1* flank |
| pNR | GTATTGTTGTGAGGGCGGCATGGTGAGTGAAGGTCTGTGCTTG |
| GFP-1F | TTTCGTAGGAACCCAATCTTCAAAATGGTGAGCAAGGGCGAGGAGCTG | For *GFP-FgNEO1* fusion construct generation |
| GFP-2R | CTTGTACAGCTCGTCCATGCCGAGAGTGAT |
| GFP-FgNEO1-1F | ATCACTCTCGGCATGGACGAGCTGTACAAGATGCCGCCCTCACAACAATACCGCCCTTCC |
| GFP-FgNEO1-2R | CACCACCCCGGTGAACAGCTCCTCGCCCTTGCTCACTTAACGATTTTGAACCTTCCTGTA |
| FgNEO1-1F | GACACAGACTCCAGTTCGTCAA | For *FgNEO1* qRT-PCR analysis |
| FgNEO1-2R | CATCACCAACCTTCAAGTCCTT |
| pFgNIA1-1F | ATGATTATCGCAACGCACCTGA | For *pFgNIA1* qRT-PCR analysis |
| pFgNIA1-2R | TAGAACGCTCCAATACATGTCC |
| Actin-1F | GTCAGTGCGGTAACCAAATCG | For *FgACTIN* qRT-PCR analysis |
| Actin-2R | CTCAGAGGTGCCGTTGTAAAC |
| Fg28s rDNA-1F | CGAGTTGTAATTTGTAGAGGATG | For *Fg*28srDNA qRT-PCR analysis |
| Fg28s rDNA-2R | GATCACTCTACTTGTGCGCTATCG |
| TaActin-F | CACTGGAATGGTCAAGGCTG | For *TaACTIN* qRT-PCR analysis |
| TaActin-R | CTCCATGTCATCCCAGTTG |
